# Effects of Elaidic Acid on Lipid Metabolism in HepG2 Cells, Investigated by an Integrated Approach of Lipidomics, Transcriptomics and Proteomics

**DOI:** 10.1371/journal.pone.0074283

**Published:** 2013-09-13

**Authors:** Lone Vendel Nielsen, Toke P. Krogager, Clifford Young, Carla Ferreri, Chryssostomos Chatgilialoglu, Ole Nørregaard Jensen, Jan J. Enghild

**Affiliations:** 1 Interdisciplinary NanoScience Center, iNANO, Aarhus University, Aarhus, Denmark; 2 Center for insoluble protein structure, InSPIN, at the Department of Molecular Biology and Genetics, University of Aarhus, Aarhus, Denmark; 3 Department of Biochemistry and Molecular Biology, University of Southern Denmark, Odense, Denmark; 4 I.S.O.F. — Consiglio Nazionale delle Ricerche, Bologna, Italy; 5 Department of Molecular Biology and Genetics, Aarhus University, Aarhus, Denmark; University of Basque Country, Spain

## Abstract

Trans fatty acid consumption in the human diet can cause adverse health effects, such as cardiovascular disease, which is associated with higher total cholesterol, a higher low density lipoprotein-cholesterol level and a decreased high density lipoprotein-cholesterol level. The aim of the study was to elucidate the hepatic response to the most abundant trans fatty acid in the human diet, elaidic acid, to help explain clinical findings on the relationship between trans fatty acids and cardiovascular disease. The human HepG2 cell line was used as a model to investigate the hepatic response to elaidic acid in a combined proteomic, transcriptomic and lipidomic approach. We found many of the proteins responsible for cholesterol synthesis up-regulated together with several proteins involved in the esterification and hepatic import/export of cholesterol. Furthermore, a profound remodeling of the cellular membrane occurred at the phospholipid level. Our findings contribute to the explanation on how trans fatty acids from the diet can cause modifications in plasma cholesterol levels by inducing abundance changes in several hepatic proteins and the hepatic membrane composition.

## Introduction

Despite increasing evidence of an adverse relationship between the human dietary intake of industrially produced trans fatty acid (IP-TFA) and cardiovascular disease [1], IP-TFAs are still common constituents of foodstuffs [2]. IP-TFA is in semi-solid fat produced by the partial hydrogenation of vegetable oils. The main isomer present is elaidic acid (EA), a monounsaturated C18 fatty acid containing a double bond at position 9 in the trans configuration [3,4]. A dietary intake of IP-TFA results in an adverse blood lipid profile with increased total cholesterol and low density lipoprotein-cholesterol (LDL-C) and a decreased level of high density lipoprotein-cholesterol (HDL-C) [5,6]. Meta-analyses of prospective cohort studies showed the incidence of coronary heart disease increased by 27% (95% CI=1.14-1.42) when 2 Energy% of monounsaturated fatty acids in the diets were replaced by IP-TFA [7].

Research aimed at elucidating the mechanisms behind the observed adverse effects of IP-TFA has focused on determining the levels and activities of single molecules involved in cholesterol trafficking between lipoproteins and cells [8,9]. However, the adverse health effects of IP-TFA intake is probably not caused by the altered activity of a single molecule, but rather the sum of changes in the levels and activities of many proteins. So far, omics technologies have only been applied to a limited extent, which include two studies of the hepatic response to EA. The first study used 2D-electrophoresis and investigated the hepatic response to EA in a hyperlipoproteinemic transgenic mouse, but only a low number of proteins were identified and even fewer were in response to the IP-TFA [10]. The second study, based on transcriptomics found that many genes were differentially expressed in hepatic tissue from mice fed on a high IP-TFA diet, but an appropriate high fat control group was absent, making it difficult to discriminate between effects derived from high fat and IP-TFA [11].

In this study we have applied a parallel proteomic, transcriptomic and lipidomic approach to unravel the hepatic cellular response to EA. A hepatocyte cell line (HepG2-SF) optimized for serum free conditions was used, allowing complete control of free fatty acid (FFA) supplemented medium composition and unambiguous identification of HepG2 secreted proteins. Stable isotope labeling by amino acids in cell culture (SILAC) was employed to investigate the long-term effects of incubation in medium supplemented with common dietary non-polyunsaturated C18 fatty acids, such as EA (trans∆9-C18:1), oleic acid (cis∆9-C18:1) (OA) or stearic acid (C18: 0) (SA) on the HepG2-SF protein expression. Furthermore, gene expression microarray analysis (GEMA) was applied to investigate gene expression after supplementation with EA or OA. Proliferation of the HepG2-SF cells in the variously supplemented media, along with their content of various fatty acids (FA) in the phospholipid (PL) fraction was measured. Our work contributes to the explanation of the molecular mechanisms behind the clinical findings regarding TFA intake and adverse health effects. 

## Experimental Procedures

### Cell culture

HepG2-SF cells (Cell Culture Service, cell passage 42) were propagated in serum-free medium composed of RPMI-1640 (Invitrogen), 10% SynQ (Cell Culture Service), 4 mM L-glutamine (Sigma-Aldrich), 20 U/mL penicillin (Invitrogen) and 20 µg/mL streptomycin (Invitrogen). Nunc culture flasks (75 cm^2^) were maintained at 37°C in a 5% CO_2_ humidified incubator. The cell culture medium was changed every four days, and the cells were subcultured every two weeks.

### SILAC adaptation of HepG2-SF cells

Medium was prepared using RPMI-1640 (Sigma) and SynQ (Cell Culture Service) without arginine, lysine and leucine. The medium was supplemented with leucine and lysine at final concentrations of 0.38 mM and 0.87 mM, respectively. From this medium, three different SILAC growth media were prepared containing 1.15 mM Arg (Sigma), ^13^C _6_Arg or ^13^C_6_^15^N _4_Arg (Cambridge Isotope Laboratories). The cells were cultured for five doublings and tested by matrix-assisted laser desorption/ionization MSfor full incorporation of ^13^C _6_Arg and ^13^C_6_^15^N _4_Arg prior to FFA incubation.

### Incubation of HepG2-SF cells in FFA-supplemented media for GEMA and SILAC experiments

FFA-supplemented medium was prepared as follows: FFAs in a 2:1 complex with human serum albumin (HSA) [12] were adjusted to a final concentration of 100 µM in serum free medium or SILAC medium. FFAs and lyophilic HSA were all obtained from Sigma-Aldrich.

Two 75 cm^2^ culture flasks containing 90% confluent cells were trypsinized and seeded into 12 petri dishes representing the three different FFA-supplementations with four biological replicates. Cells were allowed to attach for 24 h before 3 mL FFA-supplemented medium was added. The medium was changed on days 3 and 6. Only 1 mL of FFA-supplemented medium was added on day 6. After 24 h, the cell supernatant was aspirated and centrifuged at 1000 x *g* for 5 min.

The cleared cell supernatant was kept on ice until further processing. Cells for GEMA were trypsinized, washed three times in PBS and stored at -80 °C before RNA extraction. Cells for SILAC were washed in PBS (Invitrogen) and lysed in lysis buffer (1% NP-40 (Sigma-Aldrich), 150 mM NaCl, 50 mM Tris-HCl, pH 7.4) before storage at -20 °C.

### Assessment of HepG2-SF growth in FFA-supplemented medium by CyQuant proliferation assay

In a 96 well microtiter-plate, approximately 10000 cells were seeded per well and allowed to attach for 24 h before 300 µl of 100 µM FFA-supplemented medium was added (six replicas for each of the four experimental groups). On days 0, 2, 4 and 6, proliferation was assessed by adding 200 µl CyQuant dye in lysis buffer (Invitrogen) per well. After 5 min incubation, fluorescence (480 nm excitation, 520 nm emission) was measured using a FLUOstar Omega (BMG Labtech) until a plateau was obtained. Measurements significantly differing from *Controls* were determined using the unpaired t-test, two-tailed with a 95% confidence interval.

### PL extraction and characterization

A suspension (1 mL volume) containing at least 4 x 10^6^ cells (originating from the cell culture after thorough washings in PBS) was lysed by the addition of water and centrifuged at 14000 rpm for 40 min at 4°C. The membrane pellet was used to perform PL extraction as previously described [13]. The PL fraction was treated with 0.5 M KOH/MeOH for 10 min at room temperature, and the corresponding fatty acid methyl esters (FAMEs) were formed, extracted with *n*-hexane, and examined by gas chromatography analyses. Geometrical TFAs were recognized by comparison with standard references obtained by synthesis, as already described [14]. The amounts of the individual FAMEs were calculated as a percentage of the total measured FAME and their standard deviations calculated in Excel (Table S1). Measurements significantly differing from *Controls* were determined using the unpaired t-test, two-tailed with a 95% confidence interval. The data was normalized to the percentage of total measured FAME and plotted as histograms for the four series. Hierarchical clustering was done with the R-package *made4* (1.30.0) using the function *heatplot*.

### SILAC sample preparation

Cell medium was depleted of albumin by applying affinity chromatography on a column with a recombinant albumin-binding domain of streptococcal protein G [15]. The flow through was collected and the column regenerated using 20 mM sodium citrate, 150 mM NaCl, pH 2.5. The protein concentration of the depleted cell supernatant was determined using the Quick Start Bradford protein assay (Bio-Rad laboratories) and equal protein amounts from each replicate were pooled. The protein concentration for the cell lysates was determined by the 2D-quant kit (GE Healthcare) before the pooling of equal amounts of protein from each replicate.

For both cell lysates and for the depleted cell supernatants, the three SILAC groups were mixed in a 1:1:1 ratio. The proteins were separated by SDS-PAGE [16] and one lane for the cell lysate and another lane for the depleted supernatant was each cut into 22 bands. The proteins in each band were reduced, alkylated and in-gel digested with trypsin before being purified on a C_18_ Stage Tip (Thermo Scientific) and eluted with 10 µL of 80% acetonitrile. The samples were dried in a speed-vac and the peptides dissolved in 6 µL of 0.1% formic acid. LC-MS analysis was performed as described previously [17].

### SILAC Data analysis

Mass spectra were analyzed using MaxQuant software (version 1.0.13.13). The data were searched using Mascot (version 2.1.04, Matrix Science) against human international protein index protein sequence databases (version 3.52 or 3.69) supplemented with frequently observed contaminants and concatenated with reversed copies of all sequences. Quantification mode was set to triple encoding selecting Arg6 and Arg10. Enzyme specificity was set to trypsin. Propionamide was set as a variable modification. The maximum allowed mass deviation was initially set to 7 ppm for monoisotopic precursor ions and 0.6 Da for MS/MS peaks. A maximum of one missed cleavage and two labeled amino acids were allowed. The required false discovery was set to 1% at the peptide and protein level with the minimum peptide length set to six amino acids. A minimum of two unique peptides was required for protein identification. For quantification, two ratio counts were at least required. The MS datasets for the supernatant samples and the cell lysates were processed separately. The two datasets were merged in Excel and statistically significant regulated proteins were identified by a p-value < 0.01 (reported as “Significance (A)” by MaxQuant) and fold regulation >1.3. For redundant data resulting from the merging of datasets, the highest fold was reported and used for further analysis. Since the biological replicas were pooled, the reported p-values show the significance of a given protein regulation based on multiple peptides representing the same protein.

### GEMA using Human OneArray™

GEMA analysis was performed as described previously [17]. The full dataset have been deposited in NCBI’s Gene Expression Omnibus and are accessible through GEO Series accession number GSE34045.

### Ingenuity pathway analysis

The SILAC data and the GEMA data was imported into Ingenuity pathway analysis software (IPA) by their RefSeq identifiers. IPA core analyses were performed with default settings and with p-value < 0.01 for fold changes greater than 1.3 for SILAC and BH p-value < 0.01 for fold changes greater than 1.5 for GEMA. Categories within “Molecular and Cellular Functions” and “Disease and Disorders” were investigated. The results were exported and perturbed IPA categories were plotted in Excel with a BH p-value calculated by IPA to indicate the statistical significance of the category perturbation. Functions represented by the three most perturbed categories were manually extracted and the functions commonly perturbed in SILAC and GEMA were plotted in Excel with a BH p-value calculated by IPA for the statistical significance of the function perturbation. All regulated proteins and gene transcripts reported in the IPA category “Lipid Metabolism” were extracted (Table 1). 

**Table 1:** IPA “Lipid Metabolism” genes and proteins regulated in EvsO.

		GEMA (n=4)		SILAC (n=4)					
		EvsO		EvsO		EvsS			
HUGO gene symbol	Protein name	Fold change	p-value	Fold change	p-value	Fold change	p-value	Description	Reference*
**Cholesterol synthesis**									
ACAT2	Acetyl-CoA acetyltransferase	**2.3**	**1.37E-05**	**1.8**	**1.40E-07**	-1.1	3.74E-01	Synthesis of acetoacetyl-CoA from acetyl-CoA	[1]
CYP51A1	Lanosterol 14-alpha-demethylase	**3.4**	**5.95E-25**	-	-	-	-	Removes the 14-alpha-methyl group from the lanosterol	[2]
DHCR24	3-beta-hydroxysterol delta-24-reductase	3.0	1.27E-01	**1.5**	**1.57E-04**	1.7	4.30E-02	Reduces the ∆24 double bond of sterol intermediates	[3]
DHCR7	Delta-7-sterol reductase	**3.4**	**1.05E-03**	**2.4**	**9.14E-14**	**2.1**	**6.08E-03**	Reduces the ∆7 double bond of sterol intermediates	[4]
EBP	3-beta-hydroxysteroid-∆8,∆7-isomerase	**2.2**	**5.85E-05**	-	-	-	-	Converts 3-beta-Hydroxysteroid-∆8,∆7 to lathosterol	[5]
FDFT1	Farnesyldiphosphate farnesyltransferase	**4.1**	**6.63E-03**	**2.8**	**3.80E-18**	**2.5**	**9.89E-04**	Converts trans-farnesyldiphosphate to squalene	[6]
FDPS	Farnesylpyrophosphate synthetase	1.4	1.21E-01	**1.4**	**1.71E-03**	1.4	1.52E-01	Converts isopentenylpyrophosphate and dimethylallyl pyrophosphate to both geranyl and farnesylpyrophosphate	[7]
HMGCR	3-hydroxy-3-methylglutaryl-CoA reductase	**2.9**	**7.32E-14**	-	-	-	-	Synthesis of 3-hydroxy-3-methylglutaryl-CoA	[8]
HMGCS1	3-hydroxy-3-methylglutaryl-CoA synthase 1	**3.8**	**5.27E-08**	-	-	-	-	Synthesis of 3-hydroxy-3-methylglutaryl-CoA	[9]
HSD17B7	17-beta-hydroxysteroid dehydrogenase VII	2.3	7.58E-02	**2.0**	**3.31E-09**	**3.8**	**2.66E-06**	Synthesis of 4-alpha-methyl- 5-alpha-cholest-7-en-3-one	[10]
IDI1	Isopentenyl-diphosphate delta-isomerase	**2.9**	**8.54E-03**	**1.7**	**6.10E-06**	1.3	2.13E-01	Converts isopentenyl diphosphate to dimethylallyl diphosphate	[11]
LSS	Lanosterol synthase	1.7	7.94E-02	**2.3**	**2.99E-13**	**2.2**	**3.56E-03**	Cyclization of(3S) -2,3-epoxy-2,3-dihydrosqualene to lanosterol	[12]
MVK	Mevalonate kinase	**2.2**	**3.86E-07**	-	-	-	-	Converts mevalonic acid into mevalonate-5-phosphate	[13]
NSDHL	NAD(P)H steroid dehydrogenase-like protein	**2.7**	**6.74E-23**	**1.8**	**4.07E-07**	1.7	3.35E-02	Removal of two C-4 methyl groups in post-squalene cholesterol biosynth.	[14]
SQLE	Squalene epoxidase	**3.3**	**8.24E-11**	-	-	-	-	Epoxidation of C-C double bond of squalene to yield 2,3-oxidosqualene	[15]
**Cholesterol and FA synthesis**									
ACLY	ATP citrate lyase	1.3	7.50E-02	**2.3**	**2.13E-03**	1.9	3.73E-02	Synthesis of Acetyl-Co-A from citrate	[16]
CYB5B	Cytochrome b5, type B	1.6	1.72E-02	**1.6**	**4.15E-05**	1.9	1.35E-02	Electron doner for de novo synthesis	[17]
DLAT	Dihydrolipoamide acetyltransferase	-	-	**1.4**	**1.21E-03**	1.5	9.06E-02	Synthesis of Acetyl-CoA from pyruvat	[18]
SC5DL	Sterol C5-desaturase	**3.5**	**1.77E-10**	-	-	-	-	Conversion of lathosterol into 7-dehydrocholesterol	[19]
**FA synthesis**									
ACACA	Acetyl-CoA carboxylase	-1.3	5.07E-01	**1.4**	**2.70E-03**	1.1	3.66E-01	Rate-limiting in long-chain fatty acid biosynthesis	[20]
ACOT1	Acyl-CoA thioesterase 1	**7.1**	**0.00E+00**	-	-	-	-	Hydrolyze acyl-CoAs to the FFA and CoA	[21]
ACSL1	Acyl-CoA synthetase long-chain family, member 1	1.5	8.76E-02	**1.7**	**1.12E-05**	**2.3**	**1.94E-03**	Activation of long chain FAs	[22]
ACSL3	Acyl-CoA synthetase long-chain family, member 3	-	-	**1.8**	**1.15E-06**	**2.9**	**1.32E-04**	Activation of long chain FAs	[23]
ACSL4	Acyl-CoA synthetase long-chain family, member 4	**2.6**	**3.16E-10**	**1.6**	**4.64E-05**	1.8	2.36E-02	Activation of long chain FAs	[24]
ELOVL2	Elongation of very long chain fatty acids-like 2	**-2.7**	**1.40E-03**	-	-	-	-	Elongation of C20 fatty acids	[25]
ELOVL6	Elongation of very long chain fatty acids-like 6	**3.2**	**1.27E-15**	-	-	-	-	Elongation of PA to SA	[26]
FADS1	Fatty acid desaturase 1	**4.1**	**3.34E-04**	-	-	-	-	∆5 desaturase for the synthesis of arachidonic and eicosapentaenoic acid	[27]
FASN	Fatty acid synthase	1.2	4.23E-01	**1.9**	**1.01E-07**	2.2	1.35E-02	Conversion of acetyl-CoA and malonyl-CoA, in the presence of NADPH, into long-chain saturated FAs	[28]
HSD17B12	17-beta hydroxysteroid dehydrogenase-12	**5.2**	**8.75E-05**	**1.7**	**8.56E-06**	1.9	1.95E-02	Reduce 3-ketoacyl-CoA to 3-hydroxyacyl-CoA during the second step of fatty acid elongation	[29]
SCD	Stearoyl-CoA desaturase	**7.2**	**5.36E-04**	-	-	-	-	∆9 desaturase for the synthesis of palmitoleic and OA	[30]
**Secretion/Absorbtion of Cholesterol**									
LDLR	Low density lipoprotein receptor	**1.9**	**1.49E-09**	1.0	4.94E-01	-1.4	1.29E-01	Internalizes LDL particles	[31]
MTTP	Transfer RNA, mitochondrial, proline	**1.7**	**9.60E-04**	1.1	2.38E-01	1.0	4.67E-01	Incorporate CE into ApoB lipoprotein	[32]
PCSK9	Proprotein convertase, Subtilisin/kexin-type 9	**3.6**	**1.59E-11**	**3.1**	**8.16E-05**	**3.9**	**1.14E-04**	Regulates amount of LDLR on surface	[33]
SCARB1	Scavenger receptor, class B, member 1	**-1.7**	**1.80E-04**	1.2	3.46E-02	1.2	3.28E-01	Bidirectional transfer of C between cells and HDL	[34,35]
SOAT1	Sterol O-acyltransferase 1	**2.1**	**1.28E-08**	-	-	-	-	Esterification of Cholesterol	[36]
TFPI	Tissue factor pathway inhibitor	**2.3**	**3.22E-05**	**-3.6**	**1.16E-03**	**-3.0**	**7.24E-05**	Circulates associated with lipoproteins, regulates cholesterol levels, inhibit the extrinsic coagulation pathway,	[37,38]
**Lipid transport**									
ANGPTL3	Angiopoietin-like 3	**4.1**	**1.62E-04**	-	-	-	-	Inhibit LPL ie. inhibit clearance of VLDL TAGs	[39,40]
APOA4	Apolipoprotein A4	**2.6**	**2.06E-05**	1.8	2.18E-02	2.3	1.14E-02	Part of HDL, promotes C esterification and efflux in mice	[41,42]
APOE	Apolipoprotein E	1.8	1.61E-02	**1.3**	**7.22E-03**	**4.9**	**9.53E-06**	Part of VLDL and chylomicrons, ligand for receptors causing clearance of remnants	[43]
APOH	Apolipoprotein H	**2.2**	**3.33E-11**	-1.4	2.22E-01	-1.3	1.67E-01	Binds to phospholipid macromolecules, constituent of TAG rich lipoproteins, activates LPL	[44]
FABP1	Fatty acid-binding protein 1	**4.6**	**9.18E-08**	-	-	-	-	intracellular trafficking, lipid disposal (incl. Bile synthesis and secretion)	[45,46]
FABP5	Fatty acid-binding protein 5	**2.0**	**3.24E-03**	-2.4	2.03E-02	-1.4	1.17E-01	Mediates arachidonoyl ethanolamide signaling	[47]
LIPA	Lipase A, lysosomal acid	**2.5**	**1.51E-06**	-	-	-	-	Release of C from CE	[48]
OSTALPHA	Organic solute transporter, Alpha	**1.8**	**9.01E-04**	-	-	-	-	Bil acid transporter	[49]
SERPINA6	Serpin peptidase inhibitor, clade A, member 6	**1.9**	**4.63E-07**	1.0	4.64E-01	1.3	2.55E-01	Major transport protein for glucocorticoids and progestins in the blood	[50]
SAA4	Serum amyloid A1	**2.5**	**8.96E-04**	1.9	1.44E-02	**4.4**	**3.16E-05**	Constitutive apolipoprotein of HDL	[51]
**TAG metabolism**									
DGAT1	Diacylglycerol O-acyltransferase 1	**1.5**	**7.50E-04**	1.1	1.55E-01	1.1	3.66E-01	Catalyzes the terminal and only committed step in TAG synthesis by using DAG and fatty acyl CoA as substrates	[52]
DGAT2	Diacylglycerol O-acyltransferase 2	**1.6**	**3.97E-06**	-	-	-	-	Catalyzes the terminal and only committed step in TAG synthesis by using DAG and fatty acyl CoA as substrates	[53]
PNLIPRP2	Pancreatic lipase-related protein 2	**1.5**	**1.10E-07**	-	-	-	-	Hydrolysis of TAG into DAG and subsequently into MAG and FFA	[54]
PNPLA3	Patatin-like phospholipase domain-containing protein 3	**2.1**	**1.27E-05**	-	-	-	-	Hydrolysis of TAG	[55] [56]
**PL metabolism**									
ACER3	Alkaline ceramidase 3	**-1.7**	**2.19E-03**	-	-	-	-	Hydrolysis of long-chain unsaturated ceramides to generate sphingosine	[57]
ALOX12	Arachidonate 12-oxireductase	**-1.8**	**5.49E-03**	-	-	-	-	Introduces a molecular oxygen into the C-12 position of arachidonic acid to produce 12(S)-hydroperoxy-5,8,10,14-eicosatetraenoic acid	[58]
GGT5	gamma-glutamyltransferase 5	**-1.7**	**5.48E-03**	-	-	-	-	Converts leukotriene C4 to leukotriene D4	[59]
GPX4	Glutathione peroxidase 4	**1.8**	**9.07E-03**	-	-	-	-	Reduces phospholipid hydroperoxides	[60]
LIPH	Lipase H	**1.7**	**1.52E-04**	-	-	-	-	Synthesis of 2-acyl lysophosphatidic acid	[61]
LPIN1	Lpin1	**3.1**	**4.16E-07**	-	-	-	-	Catalyzes the dephosphorylation of PA to yield diacylglycerol and inorganic phosphate	[62]
LPCAT2	Lysophosphatidylcholine acyltransferase 2	**-1.7**	**3.37E-03**	-	-	-	-	Biosynthesis of PAF. Glycerophospholipid precursors from arachidonyl-CoA and lysophosphatidylcholine	[63]
PCYT2	Phosphat cytidylyltransferase 2	**6.2**	**2.51E-03**	-	-	-	-	Catalyzes the formation of CDP-ethanolamine from ethanolamine	[64]
PIK3C3	Phosphatidylinositol 3-kinase class III	**1.8**	**3.02E-05**	-	-	-	-	formation of phosphatidylinositol 3-phosphate, Involved in the transport of lysosomal enzyme precursors to lysosomes	[65]
SGPL1	Sphingosine-1-phosphate lyase 1	**2.0**	**1.25E-04**	-	-	-	-	Hydrolysis of sphingosine-1-phosphate to sphingosine	[66]
**Bile acid biosynthesis and secretion**									
AKR1C4	Aldo-keto reductase family 1, member C4	**3.1**	**1.78E-05**	-	-	-	-	Catalyzes an NAD(P)-dependent reversible oxidation of the 3-alpha-hydroxy group of various steroids and functions in the metabolism of steroid hormones and bile acids	[67,68]
DBI	Diazepam binding inhibitor	**2.6**	**1.61E-03**	-	-	-	-	Mediates the feedback regulation of pancreatic secretion and the postprandial release of cholecystokinin (bile release)	[69]
NPC2	NPC2 gene	**2.8**	**8.41E-03**	-	-	-	-	Mediates biliary cholesterol efflux through ABCG5/G8	[70]
SULT2A1	Sulfotransferase family 2A	**1.8**	**3.44E-05**	-	-	-	-	Sulfonation of bile acid	[71]
**Regulation of lipid metabolism**									
ADIPOR1	Adiponectin receptor 1	**1.6**	**4.58E-03**	-	-	-	-	Receptor for adiponectin, mediate FA oxidation, glucose uptake, PPARalpha ligand activities,	[72]
AKT2	V-akt murine thymoma viral oncogene homolog 2	**-1.9**	**2.03E-04**	-	-	-	-	Signaling, insulin responsive, FA oxidation	[73]
BRCA1	Breast cancer 1 gene	**2.0**	**1.40E-03**	-	-	-	-	Binds to phosphorylated ACACA preventing enzyme activity	[74]
CEBPA	CCAAT/enhancer-binding protein alpha	**3.7**	**2.01E-03**	-	-	-	-		[75]
DLK1	Delta, Drosophila, Homolog-like 1	**4.8**	**2.18E-12**	-	-	-	-	Regulator of adipogenesis	[76]
IDE	Insulin-degradeíng enzyme	**1.6**	**9.23E-03**	1.0	3.77E-01	-1.3	2.65E-01	Linking to lipid metabolism throuht degradation of insulin	[77]
NPPA	Natriuretic Peptide Precursor A	**-1.7**	**4.90E-07**	-	-	-	-	Regulates lipid catabolism and enhances energy dissipation through AMPK activation	[78]
SCAP	SREBP cleavage-activating protein	**-1.7**	**5.20E-03**	-	-	-	-	Involed in cholestrol sensing and cleaves SREBFs to form the active nuclear SREBFs.	[79,80]
SREBF2	Sterol regulatory element-binding protein-2	**2.3**	**3.62E-03**	-	-	-	-	In its active form regulates transcription of genes contianing sterol regulatory elements	[79,81]
ZNF202	Zinc finger protein 202	**2.0**	**1.50E-03**	-	-	-	-	transcriptional repressor of several proteins in lipid metabolism and efflux	[82]
**Energy production**									
ATP5A1	ATP synthase, H+ transporting, mitochondrial F1 complex, alpha subunit 1	**1.6**	**3.19E-04**	1.0	4.16E-01	1.1	3.96E-01	Oxidative phosphorylation	[83]
ATP5B	ATP synthase, H+ transporting, mitochondrial F1 complex, beta subunit 1	**1.5**	**4.87E-04**	1.0	4.65E-01	1.0	4.71E-01	Oxidative phosphorylation	[84]
DCI	Dodecenoyl-CoA delta isomerase	**1.5**	**2.55E-03**	1.1	3.05E-01	1.6	6.28E-02	Beta-oxidation of unsaturated FA with double bonds at odd positions	[85]
**Vitamin metabolism**									
ALDH1A1	Aldehyd dehydrogenase 1 family, member 1A	**1.7**	**6.33E-04**	1.5	7.83E-02	-2.0	4.66E-02	Oxidation of both all-trans- and 9-cis-retinal. May play a vital role in the formation of retinoic acid. Detoxification of peroxidic aldehydes produced by ultraviolet light absorption	[86,87]
ALDH1A3	Aldehyd dehydrogenase 1 family, member 3A	**-1.7**	**1.64E-05**	-	-	-	-	Role in detoxifi- cation of peroxidic aldehydes.	[88]
AOX1	Aldehyde oxidase 1	**1.7**	**4.88E-03**	-	-	-	-	AOX1 is involved in retinoic acid synthesis	[89]
DHRS3	Short-chain dehydrogenases/reductases family, member 3	**1.9**	**1.76E-06**	-	-	-	-	Generating storage forms of retinol (retinal to retinol)	[90,91]
**Other**									
CD9	CD9 antigen	**2.0**	**4.18E-04**	-	-	-	-	Undegoes acylation with palmitic acid	[92]
F2	Coagulation factor II	**2.0**	**5.89E-03**	-1.7	1.20E-01	-1.5	7.64E-02	Converts fibrinogen to fibrin for blood clot formation	[93]
GNAQ	Guanine nucleotide-binding protein, Q polypeptide.	**-1.7**	**3.69E-03**	-	-	-	-	G-alpha-q is the alpha subunit of one of the heterotrimeric GTP-binding proteins that mediates stimulation of phospholipase C-beta	[94]
GNE	UDP-N-acetylglucosamine 2-epimerase/N-acetylmannosamine kinase	**2.5**	**1.53E-04**	-	-	-	-	GNE is the rate-limiting enzyme in the sialic acid biosynthetic pathway	[95]
HPX	Hemopexin	**2.0**	**2.11E-07**	-	-	-	-	Binds one heme with high affinity and transports it to hepatocytes for salvage of iron. Protects LDL from oxidation	[96,97]
HSD17B2	17-beta-hydroxysteroid dehydrogenase II	**1.9**	**1.90E-03**	-	-	-	-	Catalyzing the interconversion of testosterone and androstenedione, as well as estradiol and estrone	[98]
KCNMA1	Potassium channel, calcium activated, large conductance, subfamily M, alpha member	**-1.7**	**1.02E-06**	-	-	-	-	Genome wide association study identifies KCNMA1 contributing to human obesity	[99]
KNG1	Kininogen 1	**3.4**	**8.56E-28**	-	-	-	-	Contain the bradykinin peptide which upon release is a potent inflammatory mediator that causes vasodilation and enhanced capillary permeability, induces pain, and stimulates production of nitric oxide and prostacyclin	[100]
LPP	Lipoma preferred partner	**2.4**	**2.28E-03**	-	-	-	-	Involved in lipoma	[101]
PHGDH	Phosphoglycerate dehydrogenase	**1.8**	**1.67E-03**	-1.1	2.25E-01	-1.4	1.98E-01	transition of 3-phosphoglycerate into 3-phosphohydroxypyruvate in serine biosynthesis	[102]
PPP1R3C	Protein phosphatase-1, regulatory subunit 3C	**3.0**	**8.78E-18**	-	-	-	-	glycogen-targeting subunit for PP1, Activates glycogen synthase, reduces glycogen phosphorylase activity and limits glycogen breakdown.	[103]
PPT1	Palmitoyl-protein thioesterase 1	**4.9**	**1.50E-13**	-	-	-	-	Removes thioester-linked fatty acyl groups such as palmitate from modified cysteine residues in proteins or peptides during lysosomal degradation. Involved in neurodegenerative disease INCL through altered cholesterol metabolism.	[104]
PRPF19	Pre-mRNA processing factor 19, homolog of *S. Cerevisiae*.	**1.6**	**1.98E-03**	1.0	3.57E-01	-1.1	4.18E-01	Found associated with lipid droplets in mice. Plays a role in DNA double-strand break (DSB) repair and pre-mRNA splicing reaction	[105,106]
SCNN1A	Sodium channel, non-voltage-gated 1 alpha subunit	**1.8**	**3.66E-03**	-	-	-	-	association with fasting insulin levels, hypertension related	[107,108]
SERPINC1	Serpin peptidase inhibitor, clade C, member 1 (Antitrombin)	**2.3**	**8.29E-06**	1.2	2.52E-01	1.2	3.12E-01	regulates blood coagulation cascade	[109]
SERPINE1	Serpin peptidase inhibitor, clade E, member 1 (Plasminogen activator inhibitor 1)	-	-	2.2	3.66E-03	1.4	1.52E-01	regulator of fibrinolysis. Binds to lipoprotein receptor-related protein (LRP) for degradation.	[110,111]
SMEK2	Suppressor of MEK1, Homolog of Dictyostelium 2	**2.3**	**5.24E-04**	-	-	-	-	involved in responsivenes to dietary cholesterol and regulation of liver triglyceride (TG) levels in rats	[112]
SNTB1	Syntrophin beta-1	**-1.7**	**7.51E-05**	-	-	-	-	involved in responsivenes to dietary cholesterol and regulation of liver TG levels in rats	[113]
STX12	Syntaxin-12	**2.2**	**8.00E-05**	-	-	-	-	Putative protein involved in the vesicular trafficking in insulin signaling	[114]
SUCLG2	Succinate-CoA ligase, GDP-forming, beta subunit	**2.1**	**9.53E-13**	1.1	2.97E-01	-1.0	4.69E-01	Catalyzes the GTP-dependent ligation of succinate and CoA to form succinyl-CoA	[115]
TTR	Transthyretin	**3.8**	**2.00E-03**	1.2	2.23E-01	2.0	2.69E-02	Associates with HDL through ApoAI. Thyroid hormone-binding protein, serum and cerebrospinal fluid (CSF) protein that transports holo-retinol-binding protein (RBP; 180250) and thyroxine (T4).	[116,117]
WWOX	WW-domain-containing oxidoreductase	**-1.8**	**8.39E-03**	-	-	-	-	Influences HDL-C levels	[118]
YWHAH	Tyrosine 3-monooxygenase/tryptophan 5-monooxygenase activation protein, eta isoform	**1.8**	**9.24E-04**	-	-	-	-	regulator of PDPK1	[119]

All genes/proteins were assigned manually to a function group based on the known literature. All genes and proteins were reported with the HUGO gene symbol, protein name, fold regulation, p-value and a description of the function according to a reference. Bold numbers indicates statistical significance of p < 0.01 for SILAC and BH p-value < 0.01 for GEMA. * Table references can be found in Table S4.

## Results

### Terminology

The different groups of HepG2-SF cells incubated in media supplemented with EA, OA, SA or no added FFA, they will be denoted as *Elaidic*, *Oleic*, *Stearic or Control*, respectively. Comparisons of e.g. *Elaidic* and *Oleic* will be denoted EvsO and of *Stearic* and *Control* SvsC.

### HepG2-SF proliferation in FFA supplemented medium

**Figure 1:**
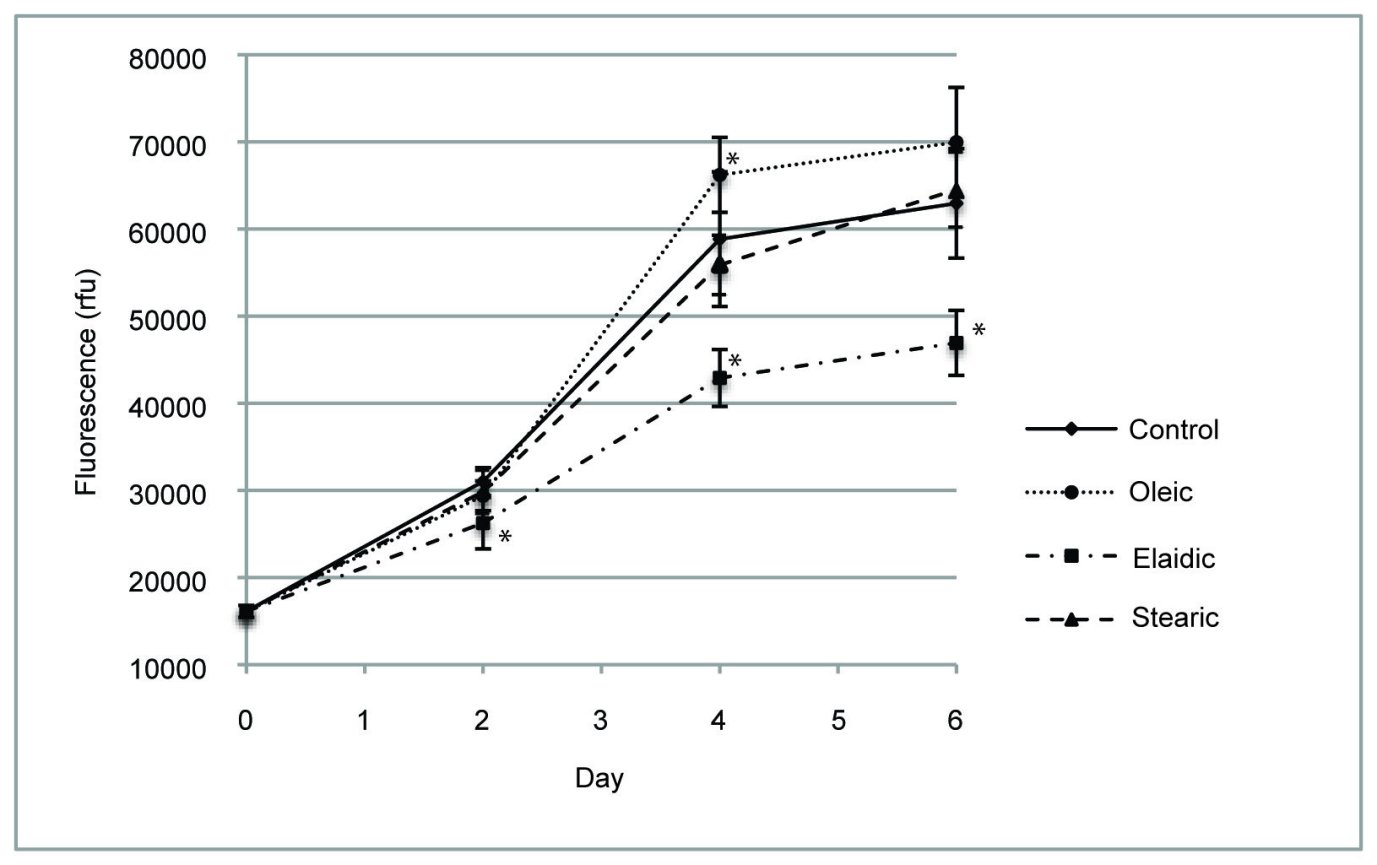
HepG2-SF cell proliferation during FA incubation. Proliferation of HepG2-SF cells in supplemented medium (100 µM FFA) was followed during six days of incubation and measured on day 0, 2, 4 and 6 by CyQuant cell proliferation assay, where fluorescence (y-axis) is a measure of cell numbers. HepG2-SF cell proliferation is differentially affected by EA, OA and SA. EA supplemented cells appear to be compromised on their growth rate when compared to *Control*, OA and SA supplemented cells. * marks measurements significantly differing from controls using the unpaired t-test, two-tailed with a 95% confidence interval.

The viability of HepG2-SF cells in the presence of 100 µM FFA was measured by a CyQuant cell proliferation assay, where the level of fluorescence is proportional to the total amount of nucleic acid and thus the total number of cells. *Stearic*, *Oleic* and *Controls* have almost equal growth rates, while *Elaidic* was slightly impaired in their proliferation rate during an incubation period of six days, particularly after day 4 (Figure 1).

### Fatty acid incorporation into the PL fraction of HepG2-SF cell membranes

**Figure 2:**
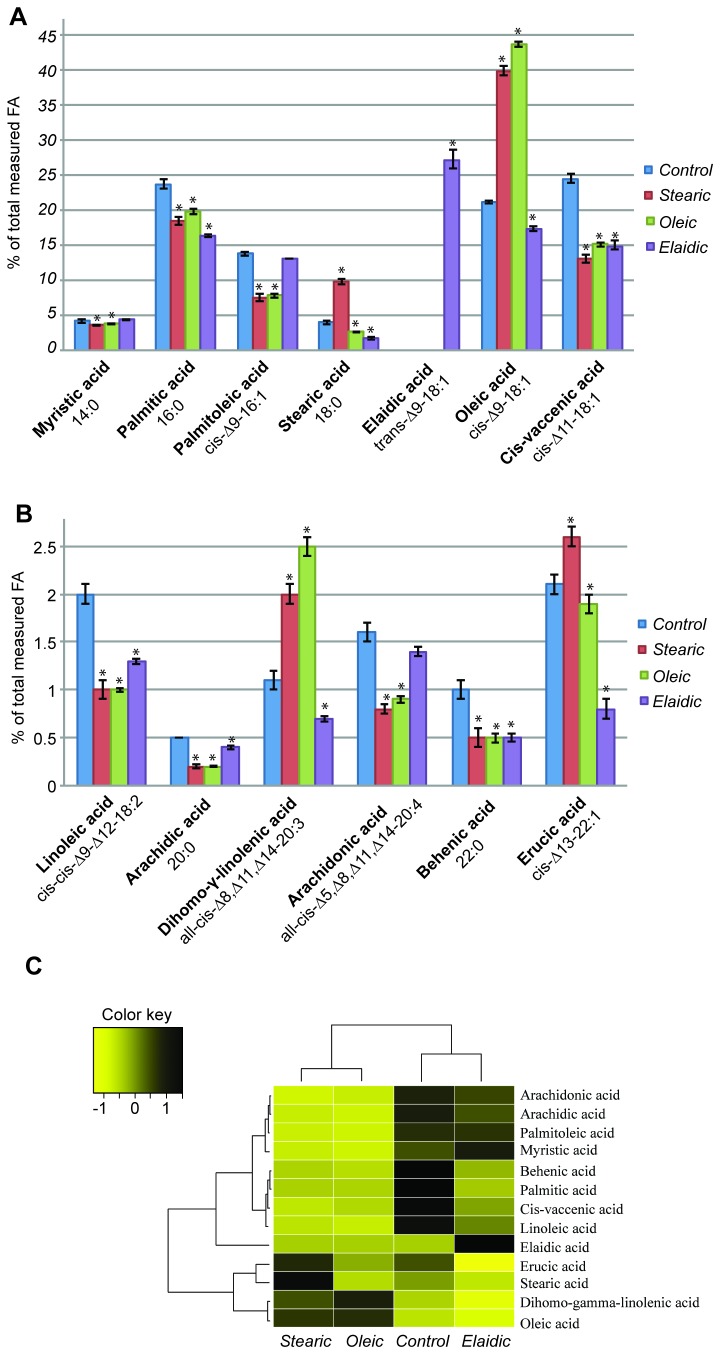
The FA composition of HepG2-SF phospholipids analyzed by gas chromatography. The composition of phospholipids from HepG2-SF cells incubated 6 days in 100 µM oleic, elaidic, or stearic acid supplemented medium were determined. Panel A and Panel B are the high and low abundant FAs present in the PLs, respectively. Each bar represents the average of three biological replicas. The y-axis is the percent of total FA methyl esters measured. The x-axis indicates the different PL FAs measured as their methyl esters. Color coding corresponds to the different supplemented FFAs. Panel C shows a heatmap representation of the distribution of FAs in PLs of the HepG2-SF cells after supplementation. The FA composition of HepG2-SF phospholipids change depending on the different supplemented fatty acids, *Stearic* and *Oleic* profiles are alike whereas the *Elaidic* FA PL profile differs from all the other groups. * marks measurements significantly differing from controls using the unpaired t-test, two-tailed with a 95% confidence interval.

The FA composition of HepG2-SF cell membrane PLs was analyzed by gas chromatography of the derived FAMEs. The most common FAs present are divided into high (Figure 2A) and low (Figure 2B) abundant FAs.

For each of the three FFA groups, a profound change in the FA composition of the plasma membrane PL was observed. The addition of SA (red bars) or OA (green bars) supplemented medium induced a two-fold increase in the SA and OA content as compared to *Control* (blue bars), from 4.0% to 9.8% and from 21.1% to 43.7%, respectively. In *Controls*, the concentration of EA was null as expected, since the double bond *trans* geometry is not naturally produced in eukaryotes. The supplementation with EA (purple bars) resulted in a 27.2% level of EA thereby indicating the effective incorporation similar to that of its cis-isomer, OA. With the high incorporation of supplemented FAs, all other FAs would expectedly decrease due to simple dilution. This dilution is observed to various degrees for almost all of the measured FAs, whereas palmitoleic acid and arachidonic acid levels seem to counteract the dilution caused by high incorporation of EA by being increased. In *Stearic* there was a significant increase in OA content compared to *Control*, with hierarchical clustering analysis revealing *Stearic* and *Oleic* have similar PL profiles (Figure 2C). To assess a possible combined effect of the PL remodeling on membrane fluidity, the average weighted melting point based on the FA composition was calculated for the membrane PLs for each of the four groups: *Oleic* (25.1°C) < *Control* (26.5°C) < *Stearic* (27.6°C) < *Elaidic* (30.7°C) (Table S1). Taken together, the PL profiles of supplemented cells suggest a profound PL remodeling in cells incubated in the presence of EA and a decreased fluidity of the membrane compartment.

### Genes found regulated after FFA supplementation

A total of 15791 transcripts were quantified across all groups in the GEMA of the cellular response. Of these, 11534 transcripts were unique based on HUGO gene symbols. After BH correction of data, a cutoff p-value <0.01 was chosen together with a fold change cutoff of 1.5. The following comparisons were considered: OvsC, EvsC and EvsO. Fewest regulations were observed in the OvsC comparison where only 30 transcripts were differentially regulated, while EvsC showed 587 differentially regulated transcripts. The highest number of regulations was in the EvsO comparison with 793 transcripts differentially regulated in response to the supplemented FFAs. A list of the transcripts differentially regulated in the three comparisons (fold change greater than1.5, Benjamini-Hochberg multiple testing corrected p-value (BH-p-value) < 0.01) appear in Table S2.

### Proteins found regulated after FFA supplementation

SILAC was used to investigate the HepG2-SF cellular response to EA, OA and SA at the protein level. The analyses resulted in the quantification of 1273 non-redundant proteins, represented by unique HUGO gene symbols, across all three groups. Comparisons involving EvsO, EvsS and SvsO were selected with fold changes greater than 1.3 and p-value<0.01 chosen as cutoff values. The highest number of regulations was observed in EvsO with a total of 88 protein regulations, while EvsS showed 76 protein regulations and SvsO showed 79 protein regulations (Full MaxQuant output can be obtained by contacting corresponding author).

### Comparison of SILAC and GEMA data

**Figure 3:**
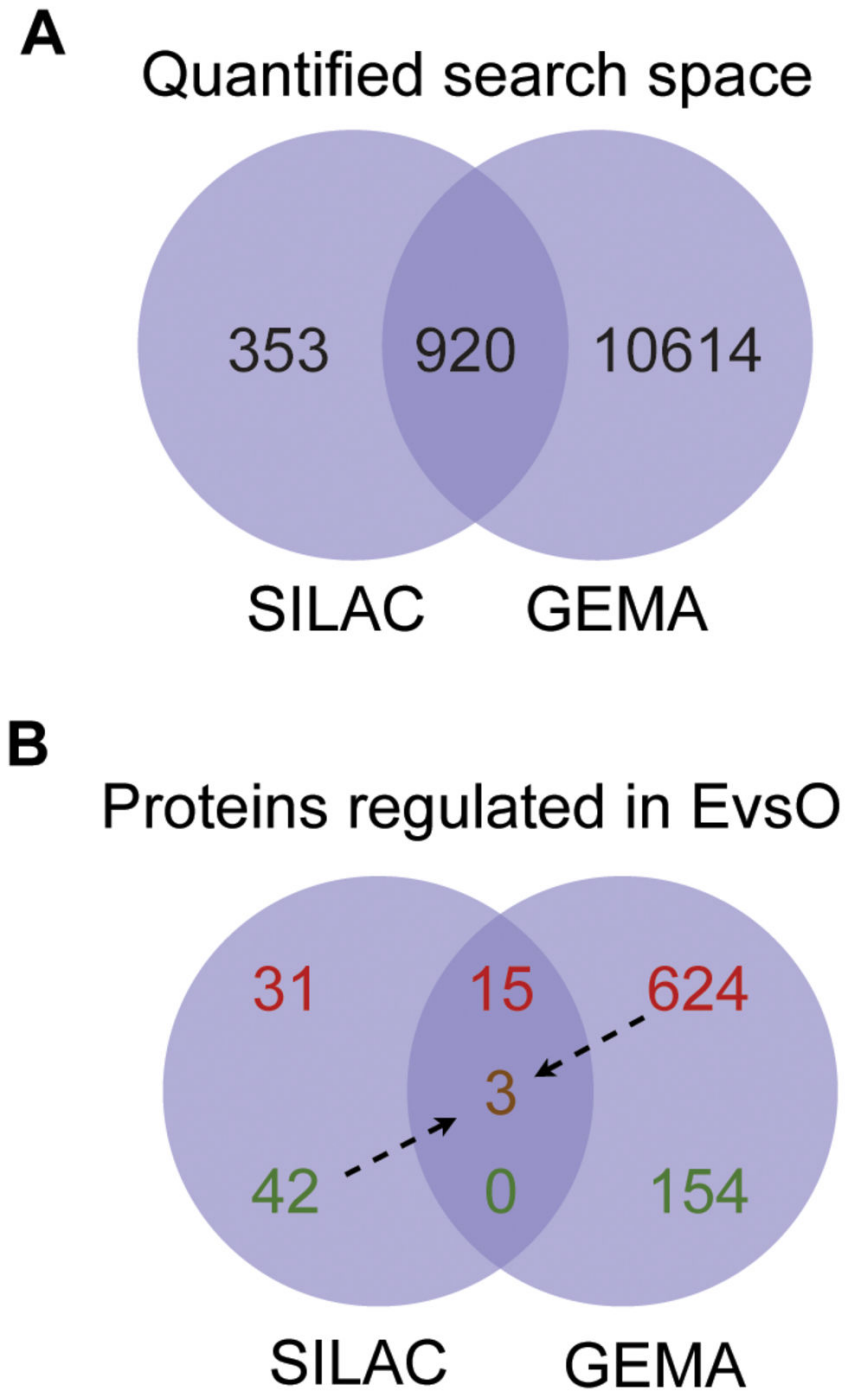
Search space comparison of genes and proteins for the comparison of EvsO differentially expressed genes/proteins in SILAC and GEMA. Panel **A**. Comparison of search spaces containing all genes/proteins quantified in SILAC and GEMA analyses. Panel **B**. Numbers of differentially expressed genes/proteins in the EvsO comparison according to method (SILAC fold change >1.3, p-value <0.01; GEMA fold change >1.5, BH-p-value <0.01). Red numbers indicate up-regulations while green indicates down-regulations. The brown number indicates genes/proteins found down-regulated in SILAC while up-regulated in GEMA. In both GEMA and SILAC, 7% of the genes/proteins in the respective search spaces were found to be regulated in the EvsO comparison.

The combined search space for SILAC and GEMA contained 11887 unique gene transcripts and proteins with HUGO gene symbols. The overlapping search space for the two methods contained 920 quantified gene transcripts/proteins (Figure 3A). Based on this combined search space, 866 transcripts/proteins were found to be differentially regulated in the EvsO comparison by SILAC and/or GEMA (Figure 3B), where 15 of these were determined by both SILAC and GEMA to be up-regulated, while no entries were down-regulated mutually by SILAC and GEMA. Three transcripts/proteins were found down-regulated by SILAC but up-regulated by GEMA. From their respective search spaces of proteins and transcripts, both SILAC and GEMA found 7% of these to be regulated in *Elaidic* when compared to *Oleic*.

### Ingenuity pathway analysis (IPA) on datasets obtained by SILAC and GEMA

The quantified proteins and gene transcripts found by SILAC and GEMA were subjected to IPA analysis. The IPA network eligible molecules included 1270 proteins quantified in SILAC and 10416 transcripts quantified in GEMA. Of the network eligible molecules, 85 proteins revealed by SILAC analysis were differentially regulated in the EvsO comparison, whereas the number of differentially regulated genes found by GEMA was 647.

**Figure 4:**
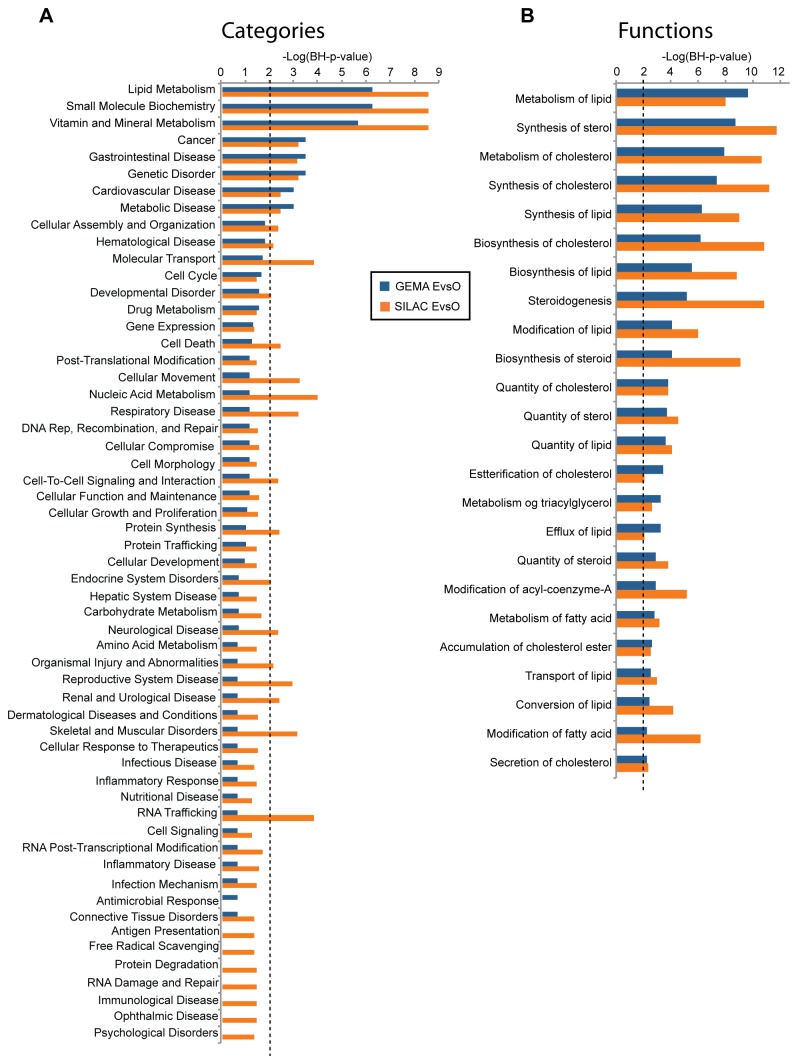
IPA categories and functions specifically perturbed by EA as compared to OA, comparing GEMA and SILAC data. **Panel** **A** displays perturbed IPA categories. The y-axis reports the significances of the perturbation and is plotted as minus log of the BH multiple testing corrected p-value calculated by IPA based on the specific data set. **Panel** **B** displays the 24 significantly perturbed functions found in the top three IPA categories for both SILAC and GEMA. For both panels, the dotted line is the threshold BH-p-value 0.01, with orange bars representing the GEMA data and the blue bars representing the SILAC data.

Proteins and transcripts that were differentially regulated in the EvsO comparison were analyzed separately by IPA in the areas of “Molecular and Cellular functions” and “Disease and Disorders”. The top three categories significantly affected in *Elaidic* when compared to *Oleic* were “Lipid Metabolism”, “Small Molecule Biochemistry” and “Vitamin and Mineral Metabolism” (Figure 4A). The top 24 functions common to both GEMA and SILAC data included in the three categories contained 118 unique genes/proteins (Figure 4B). The affected functions were mainly synthesis/metabolism of cholesterol/sterols together with more general functions related to lipid metabolism, with the 104 regulated proteins/transcripts from the Lipid Metabolism category divided into functions based on manual assignment and described in detail (Table 1).

For the SILAC analysis we also cultured HepG2-SF cells in media supplemented with SA to enable EvsS and SvsO comparisons. The affected IPA categories revealed that EvsO (blue bars) and EvsS (yellow bars) largely perturbs the same categories, while the SvsO (red bars) comparison revealed relatively few affected IPA categories that also possessed –Log(BH p-value) higher than 2 (Table S3). From the 76 proteins significantly regulated in EvsS, 27 proteins were also found in EvsO. EvsS regulation data are shown in Table 1 for the proteins/gene transcripts already identified in the IPA category “Lipid Metabolism” for the EvsO comparison. Additional statistically significant protein regulations related to the functions of “Lipid Metabolism” were found in EvsS. These were: Glutathione S-transferase A2, GSTA2; Adipose differentiation-related protein, ADFP; Apolipoprotein B, APOB; apolipoprotein M, APOM; acyl-CoA synthase short-chain family member 3, ACSS3 and prosaposin, PSAP. An integrated part of IPA is “Canonical Pathways” from the KEGG database, where the KEGG pathway “Biosynthesis of Steroids” was significantly perturbed (p-value < 0.05) in *Elaidic* when compared to *Oleic*. This was observed for both SILAC and GEMA. 

## Discussion

The aim of this study was to use transcriptomic, proteomic and lipidomic approaches to elucidate cellular responses that contribute to the observed clinical effects of TFA intake. It is important to unravel the mechanisms that contribute to the adverse effects of TFA. The intake of TFAs may increase the total cholesterol:HDL-C and LDL-C:HDL-C ratios [5,6] and increase the risk of cardiovascular diseases [1].

HepG2 SF cells show impaired proliferation rate when supplemented with EA, whereas cellular proliferation during OA supplementation does not differ from *Controls*. However, EA is not the only TFA present in human diet, several other TFAs are present at similar or lower amounts and these may also contribute to the negative health effects observed in relation to TFA containing diets. Analysis of platelets from patients with coronary artery disease showed a correlation of both EA and trans-10-C18:1 with disease risk [18], whereas our own analysis of the HepG2 response to vaccenic acid (trans-11 C18: 1) shows less pronounced effect on cell proliferation than EA (data not shown). The effect of TFA mixtures on HepG2 cells and on humans will thus most likely depend on the composition and will be difficult to predict. OA have been reported to reverse stearic acid-induced inhibition of cell growth in human aortic endothelial cells [19] and to attenuate the effect of trans-10, cis-12 conjugated fatty acid mediated inflammatory gene expression in human adipocytes [20]. Thus, if a mixture of OA and EA had been used, both the proliferative, transcriptomic, lipidomic and proteomic response may be affected, and the adverse consequence of TFA would be more difficult to investigate. In this study, we have investigated the hepatocellular response to supplementation with single fatty acids to focus on specific responses and especially to compare EA to OA.

From the 11534 transcripts analyzed by GEMA and 1273 proteins analyzed by SILAC, we report 866 gene transcripts and/or proteins with statistically significant altered expression between EA and OA supplemented HepG2-SF cells after 7 days of incubation. The IPA software found that “Lipid Metabolism”, “Small Molecule Biochemistry” and “Vitamin and Mineral Metabolism” were the top ranked perturbed categories, but since the three categories contain an almost identical ensemble of genes/proteins, only the “Lipid metabolism” category was explored further (Figure 4A and Table 1). The molecular functions found perturbed within this category related to lipid metabolism in general and more specifically to cholesterol synthesis/metabolism and FA synthesis/metabolism (Figure 4B and Table 1).

SA was included in the experimental setup to investigate if the effect observed for EA could be attributed to the high FA melting point. An investigation of the regulated proteins in SvsO with IPA found only a few and weakly perturbed IPA categories (Table S3), indicating a common effect for SA and OA on known molecular functions. The common effects of SA and OA can be attributed to a possible conversion of SA to OA by SCD (all abbreviations for protein names used in the following text can be found in Table 1). From the IPA analysis it was also observed that the same categories were perturbed in both EvsO and EvsS comparisons, namely “Lipid Metabolism”, “Small Molecule Biochemistry” and “Vitamin and Mineral Metabolism”. Many of the enzymes responsible for the direct cholesterol synthesis were statistically significant regulated or trending similarly in EvsO and EvsS, indicating that EA is cholesterologenic compared to both SA and OA (Table 1). Selected proteins and possible implications of the response in *Elaidic* as compared with *Oleic* are discussed below.

### The cholesterol sensing machinery may be affected

The expression of enzymes responsible for cholesterol synthesis depends on the transcription factor SREBP2 and to a lesser extent SREBP1a [21]. Mammalian cells sense the intracellular level of cholesterol through SCAP and Insig1/2. SREBPs interact with SCAP and at high cholesterol levels, the SREBP:SCAP complex is retained in the endoplasmic reticulum, ER, through its interaction with Insig1/2. At low levels of cholesterol, the SREBP:SCAP complex dissociates from Insig1/2 and translocates from the ER to the Golgi. After two proteolytic cleavages, the active transcription factor, nuclear SREBP, is formed. Upon further translocation to the nucleus nuclear SREBP activates transcription of the cholesterol synthesizing genes [22]. Upon EA supplementation, we observed SREBP2 to be statistically significant up-regulated and SCAP to be statistically significant down-regulated, while Insig2 showed a trend towards up-regulation (but was not found to be statistically significant). These changes indicate an alteration in the cholesterol sensing and synthesis inducing machinery (Table S2).

### Essentially all proteins involved in cholesterol synthesis are up-regulated by EA

Besides the acetyl-CoA synthesizing enzymes ACLY and DLAT, 16 enzymes in the cholesterol synthesis pathway were found statistically significant up-regulated, ranging from 1.5 to 4.1 fold, including the rate-limiting enzyme HMGCR, which shows an up-regulation of 2.9 fold in GEMA. Other studies have reported increased cholesterol synthesis, export and total cholesterol in HepG2 cell supernatants as a response to long-term supplementation with EA compared to OA [23]. We observed that the expression of essentially all of the enzymes responsible for cholesterol synthesis was increased after seven days of incubation with EA, verifying a sustained effect on cholesterol synthesis.

### FA synthesis is affected

High levels of cholesterol are cytotoxic for cells [24] and thus excess cholesterol has to be esterified with activated FAs for export or storage. ACACA is the rate-limiting enzyme in the synthesis of long chain FA [25] and both ACACA and FAS (which is responsible for the synthesis of palmitic acid (PA) from acetyl-CoA) were significantly up-regulated. ELOVL6 was also significantly up-regulated and is responsible for elongation of PA to SA. SCD desaturates both PA and SA to yield the monounsaturated fatty acids palmitoleic acid and OA, respectively, and was found significantly up-regulated. The up-regulation of these proteins in the FA synthesis indicates that supplementation with EA initiates synthesis of fundamental FAs like PA, palmitoleic acid, SA and OA.

DGAT1 and 2 synthesizes tri-acylglycerol (TAG) from di-acylglycerol (DAG) and CoA-activated FAs, which is the terminal and only committed step in TAG synthesis. Transcripts for the two proteins were found up-regulated which could point to a cytosolic accumulation of FAs as TAGs in lipid droplets [26]. PNPLA3 was upregulated, and with new studies showing its physiological role involving the synthesis of TAG [27], it supports the regulations of DGAT1 and 2. In the PL analysis (Figure 2A), it was observed that the combined amount of PA and palmitoleic acid in *Elaidic* was higher compared to both *Oleic* and *Stearic*, which could be explained by an increased FA *de novo* synthesis. The observation of a lower PA level and a higher level of palmitoleic acid in *Elaidic* than in *Oleic* and *Stearic* could be explained by an increase in desaturase activity as a consequence of the observed increase in SCD expression. This could also explain the low level of SA and lower than expected dilution of OA in *Elaidic* PLs.

### Cholesterol ester synthesis and Export seems increased

It has been shown in mice that SCD is essential for the synthesis of cholesterol esters (CE) because it provides the unsaturated FAs for the CE synthesis from cholesterol [28]. ACSL1, 3 and 4, which are needed for the activation of FFAs with CoA, were all found significantly up-regulated together with SOAT1, which esterifies free cholesterol with activated FFAs [29], and MTTP, which is capable of incorporating CE into ApoB lipoproteins. The accumulation of CE should decrease CE synthesis, but this can be counteracted by MTTP *via* incorporating CEs into ApoB100 containing very low density lipoprotein (VLDL) for secretion [30]. We did not find any ApoB100 expression increases in either GEMA or SILAC, while an earlier study reported no change in the ApoB content of cell supernatants from HepG2 cells treated with EA or OA [23]. The same study reported that the cholesterol content in secreted VLDL, LDL and HDL increased 43%, 70% and 34%, respectively, upon EA supplementation.

### Bile synthesis

Another route of cholesterol export is in the form of bile acids. We found several up-regulated proteins which may suggest an increased export of cholesterol into the bile. These include SULT2A1 and NPC2, which increases bile hydrophilicity by sulfonation and mediates biliary cholesterol efflux through the ABCG5/G8 transporter [31] respectively.

### Cholesterol import may be decreased by elaidic acid supplementation

Both LDL-receptor (LDLR) and PCSK9 levels increased in *Elaidic* (>3 fold in both SILAC and GEMA), most likely as a consequence of increased expression and/or activation of the transcription factor SREBP2 [32]. PCSK9 causes degradation of the LDLR upon binding, resulting in a decreased level of LDLR at the cell surface [33] and thus despite increased synthesis of LDLR, the overall level of the receptor at the cell surface may be decreased. LDLR is responsible for receptor mediated endocytosis of LDL and thus decreased LDLR would lead to decreased uptake of LDL from the surroundings. ApoB/CE containing LDL may therefore accumulate extracellularly and explain the findings by others that ApoB-LDL increases in plasma due to TFA intake [5].

SAA4 is a constitutive apolipoprotein of HDL [34] and ApoA4 is also found associated with HDL or free in plasma [35]. ApoA4 can promote cholesterol efflux from peripheral tissues and esterification into HDL via increased activity of lecithin-cholesterol acyltransferase, LCAT, extracellularly [36]. LCAT is trending towards up-regulation and we also find increased transcript levels of SAA4 and ApoA4, which may point towards an increased extracellular level of HDL and an increased capacity for extracellular cholesterol. The receptor SCARB1 mediates the bidirectional transfer of cholesterol and cholesterol esters between liver cells and HDL [37,38]. As SCARB1 transcription is decreased in *Elaidic*, the hepatocyte reuptake of secreted cholesterol in the form of cholesterol esters could be compromised.

Taken together, our study supports an increase in total cholesterol:HDL-C and LDL-C:HDL-C ratios due to TFA intake. The EA-induced changes in protein/gene transcript levels indicate an increase in the synthesis of cholesterol together with an increased extracellular capacity for esterified cholesterol in both HDL and LDL particles. Together with the possibility of increased level of LCAT and increased CETP activity [9], cholesterol may be accumulating in LDL particles.

### Indications of PL de novo synthesis and remodeling

Several transcripts for enzymes involved in the *de novo* synthesis and remodeling of PLs were found statistically significant regulated in the EvsO comparison. PCYT2, LPIN1 and LIPH are enzymes involved in PL class shift and general FA remodeling of PLs and all were found to be up-regulated. PCYT2 is the main regulatory enzyme for the *de novo* synthesis of phosphatidylethanolamine (PE) from DAG and CDP-ethanolamine (Kennedy pathway) [39,40]. EA supplementation has previously shown to increase the PE content of cell membranes while the content of phosphatidylcholine (PC) remained unchanged [41]. LPIN1 and LIPH hydrolyze phosphatidic acid to DAG and lyso-phosphatidic acid. Furthermore, the enzyme DGKA, responsible for the reverse reaction of LPIN1 [42], trends towards a down-regulation (1.4 fold down, BH-p-value= 0.0448), all together indicating a decreased level of phosphatidic acid and increased levels of DAG, which could be used for the PE synthesis. LPCAT2 [43] is statistically significant down-regulated while LCAT is trending towards up-regulation (2 fold up, BH-p-value= 0.0448), which are key enzymes in the Land’s Cycle [44] of remodeling of PC. The collective regulation of these genes indicates an increased transfer of acyl chains from PC to cholesterol and thus also contributing to the formation and accumulation of extracellular CEs. It should also be mentioned that TFA in membranes increase the calcium influx into cells [45] and that increased calcium levels activate phospholipase A2, which is essential for lipid remodeling [46].

Another aspect of cholesterol and TFA in PLs is the linear configuration of TFA, which causes the TFA-PLs to pack tightly. TFA-containing artificial PL membranes also tend to bind more cholesterol than membranes without TFA [47]. The membrane cholesterol activity (amount of free cholesterol e.g. for export) is decreased, and the function and activity of integral membrane proteins and channels can be compromised due to the more rigid membrane [45,47,48]. Cell experiments have shown that the viscosity of cell membranes increase when the culture medium was supplemented with EA [41], which is supported by our calculations of relative membrane melting points based on the FA compositions, *Oleic* (25.1°C) <*Control* (26.5°C) <*Stearic* (27.6°C) <*Elaidic* (30.7°C). Studies on model lipid membranes containing TFA also show a reduced permeability [49].

EA has been shown to be rapidly taken up, oxidized for energy production or incorporated into the sn1-position of phospholipids and thereby displacing saturated FAs. This incorporation has been observed to be stable over time [50]. Furthermore, it has been shown *ex vivo* that the physical properties of the hydrophobic core of PLs did not change upon 18% EA incorporation, corresponding to one-third to two-fifths of PC, PE and phosphatidylinositol molecules containing one molecule of EA [51]. In our study, we observed an even higher incorporation of EA (27%) into the PLs of HepG2-SF cells, resulting in an overall FA profile differing from OA and SA treated cells, with the latter two showing similar profiles (Figure 2ABC). We observed the lowest amounts of PA and SA in *Elaidic* in accordance with the previous observations that EA displaces these saturated FAs. Moreover, from figure 2ABC it is clear that monounsaturated- and polyunsaturated FA residues have been displaced as well, and overall this induces a profound change in the properties of the membrane compartment. FA supplementation induces the PL remodeling and the consequences of supplementation with a non-natural FA like EA have been elucidated by the PL profiling and omics methods used here. The results suggest further combination with targeted studies in characterizing PL classes and their flux during EA supplementation. 

## Conclusion

FA remodeling in membrane phospholipids obtained from EA supplemented HepG2 cells was compared with saturated (SA) and monounsaturated (OA) supplementations. The results were integrated with transcriptomic and proteomic data, thus allowing for a comprehensive mapping of effects on lipid biosynthesis, metabolism and membrane status. In the presented study we have found that EA clearly induces an increase in cholesterol synthesis through significant up-regulation of essentially all proteins involved. The expression of proteins involved in FA synthesis, activation and esterification to cholesterol also increased and several proteins were found regulated as a means to export newly synthesized cholesterol and FA. Our finding on the regulation of several proteins involved in cholesterol and FA metabolism contributes to the unraveling of the mechanism behind the EA induced increase in cholesterol synthesis, export and extracellular levels observed by others and the distribution in HDL and LDL particles. The underlying causes of the observed effects warrant further investigations, but our findings has led us to the hypothesis that the cellular cholesterol sensing machinery including Insig1/2 and SCAP is perturbed by the presence of EA. It has been suggested that (i), the cholesterol is trapped by TFA-PLs in membranes [47] causing a lowered cholesterol activity and thus it is inaccessible for the sensory proteins [52], or (ii), the sensory proteins do not function optimally due to increased rigidity of the membrane [47]. As a consequence of lower cholesterol activity, the cell may increase cholesterol synthesis to restore membrane free cholesterol. Alternatively, we speculate that EA could affect the cholesterol sensing proteins directly by blocking the cholesterol binding site or indirectly by causing degradation of the sensory components. Due to the resulting cytotoxicity from cholesterol accumulation, the cell responds by increasing the export of cholesterol. Increased cholesterol synthesis and export in combination with decreased uptake in terms of altered hepatic protein expressions may be the reasons for the observations in the literature where IP-TFA alters total cholesterol, increases LDL-C/HDL-C ratios and contributes to a higher risk of cardiovascular diseases. 

## Supporting Information

Table S1 **FAME analysis.**
(XLS)

Table S2 **GEMA BH corrected COE.**
(XLS)

Table S3 **Categories graph SILAC.**
(PNG)

Table S4 **Table references.**
(DOCX)
