# Supplementary figures and images for: Effects of Elaidic Acid on Lipid Metabolism in HepG2 Cells, Investigated by an Integrated Approach of Lipidomics, Transcriptomics and Proteomics
Source: PLoS One. 2013 Sep 13;8(9):e74283. doi: 10.1371/journal.pone.0074283 (PMC3772929; doi:10.1371/journal.pone.0074283)

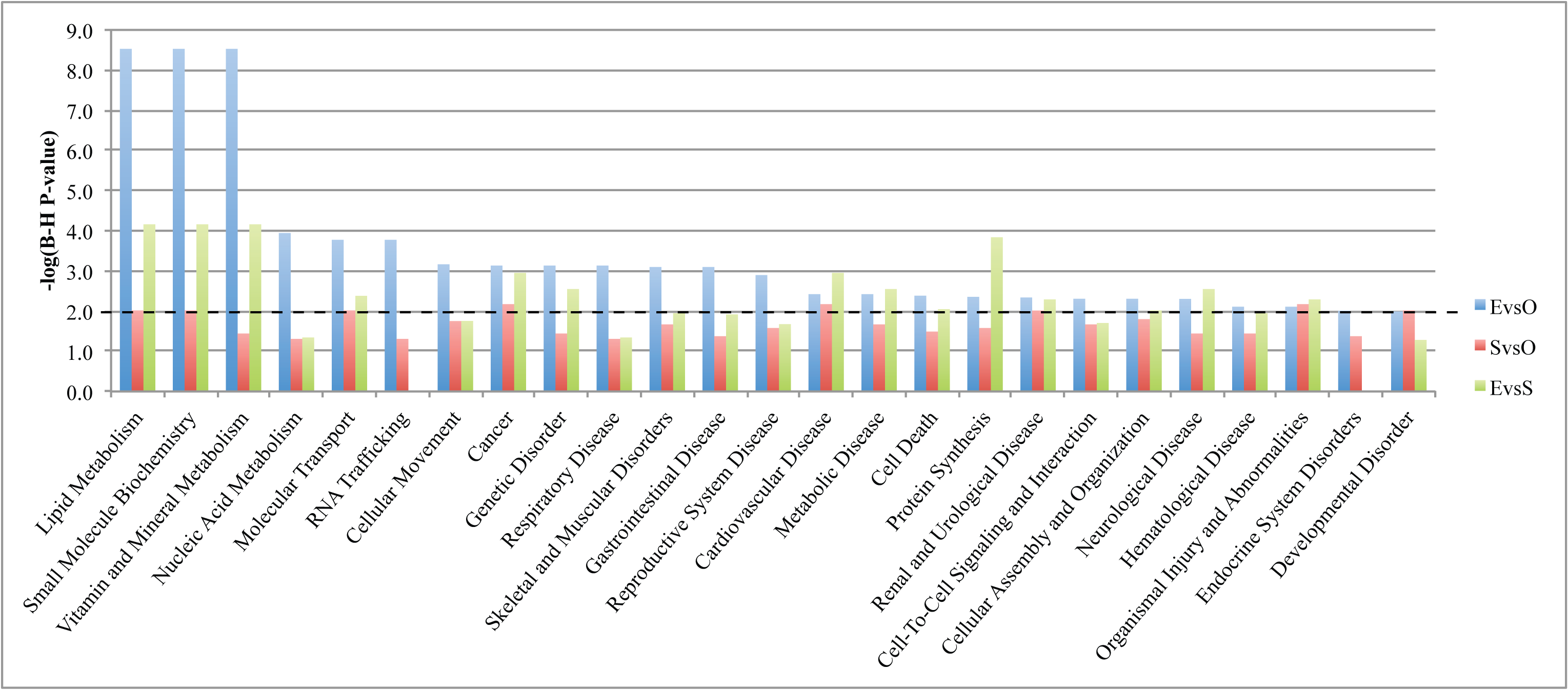

Supplement: Table S3 — Categories graph SILAC. (PNG) [file pone.0074283.s003.png]
