# Supplementary material for: Effects of Elaidic Acid on Lipid Metabolism in HepG2 Cells, Investigated by an Integrated Approach of Lipidomics, Transcriptomics and Proteomics
Source: PLoS One. 2013 Sep 13;8(9):e74283. doi: 10.1371/journal.pone.0074283 (PMC3772929; doi:10.1371/journal.pone.0074283)
Supplement: Table S4 — Table references. (DOCX) [file pone.0074283.s004.docx]

***References for table 1:***

1. Kursula P, Sikkila H, Fukao T, Kondo N, Wierenga RK (2005) High resolution crystal structures of human cytosolic thiolase (CT): a comparison of the active sites of human CT, bacterial thiolase, and bacterial KAS I. J Mol Biol 347: 189-201.

2. Stromstedt M, Rozman D, Waterman MR (1996) The ubiquitously expressed human CYP51 encodes lanosterol 14 alpha-demethylase, a cytochrome P450 whose expression is regulated by oxysterols. Arch Biochem Biophys 329: 73-81.

3. Bae SH, Paik YK (1997) Cholesterol biosynthesis from lanosterol: development of a novel assay method and characterization of rat liver microsomal lanosterol delta 24-reductase. Biochem J 326 ( Pt 2): 609-616.

4. Wassif CA, Maslen C, Kachilele-Linjewile S, Lin D, Linck LM, et al. (1998) Mutations in the human sterol delta7-reductase gene at 11q12-13 cause Smith-Lemli-Opitz syndrome. Am J Hum Genet 63: 55-62.

5. Silve S, Dupuy PH, Labit-Lebouteiller C, Kaghad M, Chalon P, et al. (1996) Emopamil-binding protein, a mammalian protein that binds a series of structurally diverse neuroprotective agents, exhibits delta8-delta7 sterol isomerase activity in yeast. J Biol Chem 271: 22434-22440.

6. Schechter I, Conrad DG, Hart I, Berger RC, McKenzie TL, et al. (1994) Localization of the squalene synthase gene (FDFT1) to human chromosome 8p22-p23.1. Genomics 20: 116-118.

7. Barnard GF (1985) Prenyltransferase from human liver. Methods Enzymol 110: 155-167.

8. Goldstein JL, Brown MS (1990) Regulation of the mevalonate pathway. Nature 343: 425-430.

9. Rokosz LL, Boulton DA, Butkiewicz EA, Sanyal G, Cueto MA, et al. (1994) Human cytoplasmic 3-hydroxy-3-methylglutaryl coenzyme A synthase: expression, purification, and characterization of recombinant wild-type and Cys129 mutant enzymes. Arch Biochem Biophys 312: 1-13.

10. Torn S, Nokelainen P, Kurkela R, Pulkka A, Menjivar M, et al. (2003) Production, purification, and functional analysis of recombinant human and mouse 17beta-hydroxysteroid dehydrogenase type 7. Biochem Biophys Res Commun 305: 37-45.

11. Hahn FM, Xuan JW, Chambers AF, Poulter CD (1996) Human isopentenyl diphosphate: dimethylallyl diphosphate isomerase: overproduction, purification, and characterization. Arch Biochem Biophys 332: 30-34.

12. Baker CH, Matsuda SP, Liu DR, Corey EJ (1995) Molecular cloning of the human gene encoding lanosterol synthase from a liver cDNA library. Biochem Biophys Res Commun 213: 154-160.

13. Hinson DD, Chambliss KL, Toth MJ, Tanaka RD, Gibson KM (1997) Post-translational regulation of mevalonate kinase by intermediates of the cholesterol and nonsterol isoprene biosynthetic pathways. J Lipid Res 38: 2216-2223.

14. Caldas H, Herman GE (2003) NSDHL, an enzyme involved in cholesterol biosynthesis, traffics through the Golgi and accumulates on ER membranes and on the surface of lipid droplets. Hum Mol Genet 12: 2981-2991.

15. Hidaka Y, Satoh T, Kamei T (1990) Regulation of squalene epoxidase in HepG2 cells. J Lipid Res 31: 2087-2094.

16. Elshourbagy NA, Near JC, Kmetz PJ, Wells TN, Groot PH, et al. (1992) Cloning and expression of a human ATP-citrate lyase cDNA. Eur J Biochem 204: 491-499.

17. Fukushima H, Grinstead GF, Gaylor JL (1981) Total enzymic synthesis of cholesterol from lanosterol. Cytochrome b5-dependence of 4-methyl sterol oxidase. J Biol Chem 256: 4822-4826.

18. Patel MS, Roche TE (1990) Molecular biology and biochemistry of pyruvate dehydrogenase complexes. FASEB J 4: 3224-3233.

19. Nishi S, Nishino H, Ishibashi T (2000) cDNA cloning of the mammalian sterol C5-desaturase and the expression in yeast mutant. Biochim Biophys Acta 1490: 106-108.

20. Lopez-Casillas F, Bai DH, Luo XC, Kong IS, Hermodson MA, et al. (1988) Structure of the coding sequence and primary amino acid sequence of acetyl-coenzyme A carboxylase. Proc Natl Acad Sci U S A 85: 5784-5788.

21. Hunt MC, Rautanen A, Westin MA, Svensson LT, Alexson SE (2006) Analysis of the mouse and human acyl-CoA thioesterase (ACOT) gene clusters shows that convergent, functional evolution results in a reduced number of human peroxisomal ACOTs. FASEB J 20: 1855-1864.

22. Parkes HA, Preston E, Wilks D, Ballesteros M, Carpenter L, et al. (2006) Overexpression of acyl-CoA synthetase-1 increases lipid deposition in hepatic (HepG2) cells and rodent liver in vivo. Am J Physiol Endocrinol Metab 291: E737-744.

23. Watkins PA, Maiguel D, Jia Z, Pevsner J (2007) Evidence for 26 distinct acyl-coenzyme A synthetase genes in the human genome. J Lipid Res 48: 2736-2750.

24. Cao Y, Traer E, Zimmerman GA, McIntyre TM, Prescott SM (1998) Cloning, expression, and chromosomal localization of human long-chain fatty acid-CoA ligase 4 (FACL4). Genomics 49: 327-330.

25. Leonard AE, Kelder B, Bobik EG, Chuang LT, Lewis CJ, et al. (2002) Identification and expression of mammalian long-chain PUFA elongation enzymes. Lipids 37: 733-740.

26. Ohno Y, Suto S, Yamanaka M, Mizutani Y, Mitsutake S, et al. (2010) ELOVL1 production of C24 acyl-CoAs is linked to C24 sphingolipid synthesis. Proc Natl Acad Sci U S A 107: 18439-18444.

27. Nakamura MT, Nara TY (2004) Structure, function, and dietary regulation of delta6, delta5, and delta9 desaturases. Annu Rev Nutr 24: 345-376.

28. Wakil SJ (1989) Fatty acid synthase, a proficient multifunctional enzyme. Biochemistry 28: 4523-4530.

29. Moon YA, Horton JD (2003) Identification of two mammalian reductases involved in the two-carbon fatty acyl elongation cascade. J Biol Chem 278: 7335-7343.

30. Miyazaki M, Kim YC, Gray-Keller MP, Attie AD, Ntambi JM (2000) The biosynthesis of hepatic cholesterol esters and triglycerides is impaired in mice with a disruption of the gene for stearoyl-CoA desaturase 1. J Biol Chem 275: 30132-30138.

31. Brown MS, Goldstein JL (1979) Receptor-mediated endocytosis: insights from the lipoprotein receptor system. Proc Natl Acad Sci U S A 76: 3330-3337.

32. Iqbal J, Rudel LL, Hussain MM (2008) Microsomal triglyceride transfer protein enhances cellular cholesteryl esterification by relieving product inhibition. J Biol Chem 283: 19967-19980.

33. Qian YW, Schmidt RJ, Zhang Y, Chu S, Lin A, et al. (2007) Secreted PCSK9 downregulates low density lipoprotein receptor through receptor-mediated endocytosis. J Lipid Res 48: 1488-1498.

34. Ji Y, Jian B, Wang N, Sun Y, Moya ML, et al. (1997) Scavenger receptor BI promotes high density lipoprotein-mediated cellular cholesterol efflux. J Biol Chem 272: 20982-20985.

35. Varban ML, Rinninger F, Wang N, Fairchild-Huntress V, Dunmore JH, et al. (1998) Targeted mutation reveals a central role for SR-BI in hepatic selective uptake of high density lipoprotein cholesterol. Proc Natl Acad Sci U S A 95: 4619-4624.

36. Brown MS, Ho YK, Goldstein JL (1980) The cholesteryl ester cycle in macrophage foam cells. Continual hydrolysis and re-esterification of cytoplasmic cholesteryl esters. J Biol Chem 255: 9344-9352.

37. Holroyd EW, Simari RD (2010) Interdependent biological systems, multi-functional molecules: the evolving role of tissue factor pathway inhibitor beyond anti-coagulation. Thromb Res 125 Suppl 1: S57-59.

38. Novotny WF, Girard TJ, Miletich JP, Broze GJ, Jr. (1989) Purification and characterization of the lipoprotein-associated coagulation inhibitor from human plasma. J Biol Chem 264: 18832-18837.

39. Shimamura M, Matsuda M, Yasumo H, Okazaki M, Fujimoto K, et al. (2007) Angiopoietin-like protein3 regulates plasma HDL cholesterol through suppression of endothelial lipase. Arterioscler Thromb Vasc Biol 27: 366-372.

40. Shimizugawa T, Ono M, Shimamura M, Yoshida K, Ando Y, et al. (2002) ANGPTL3 decreases very low density lipoprotein triglyceride clearance by inhibition of lipoprotein lipase. J Biol Chem 277: 33742-33748.

41. Lagrost L, Gambert P, Boquillon M, Lallemant C (1989) Evidence for high density lipoproteins as the major apolipoprotein A-IV-containing fraction in normal human serum. J Lipid Res 30: 1525-1534.

42. Duverger N, Ghalim N, Ailhaud G, Steinmetz A, Fruchart JC, et al. (1993) Characterization of apoA-IV-containing lipoprotein particles isolated from human plasma and interstitial fluid. Arterioscler Thromb 13: 126-132.

43. Ruiz J, Kouiavskaia D, Migliorini M, Robinson S, Saenko EL, et al. (2005) The apoE isoform binding properties of the VLDL receptor reveal marked differences from LRP and the LDL receptor. J Lipid Res 46: 1721-1731.

44. Nakaya Y, Schaefer EJ, Brewer HB, Jr. (1980) Activation of human post heparin lipoprotein lipase by apolipoprotein H (beta 2-glycoprotein I). Biochem Biophys Res Commun 95: 1168-1172.

45. Dietrich A, Dieminger W, Fuchte K, Stoll GH, Schlitz E, et al. (1995) Functional significance of interaction of H-FABP with sulfated and nonsulfated taurine-conjugated bile salts in rat liver. J Lipid Res 36: 1745-1755.

46. McArthur MJ, Atshaves BP, Frolov A, Foxworth WD, Kier AB, et al. (1999) Cellular uptake and intracellular trafficking of long chain fatty acids. J Lipid Res 40: 1371-1383.

47. Kaczocha M, Glaser ST, Deutsch DG (2009) Identification of intracellular carriers for the endocannabinoid anandamide. Proc Natl Acad Sci U S A 106: 6375-6380.

48. Warner TG, Dambach LM, Shin JH, O'Brien JS (1980) Separation and characterization of the acid lipase and neutral esterases from human liver. Am J Hum Genet 32: 869-879.

49. Seward DJ, Koh AS, Boyer JL, Ballatori N (2003) Functional complementation between a novel mammalian polygenic transport complex and an evolutionarily ancient organic solute transporter, OSTalpha-OSTbeta. J Biol Chem 278: 27473-27482.

50. Rosner W (1990) The functions of corticosteroid-binding globulin and sex hormone-binding globulin: recent advances. Endocr Rev 11: 80-91.

51. Whitehead AS, de Beer MC, Steel DM, Rits M, Lelias JM, et al. (1992) Identification of novel members of the serum amyloid A protein superfamily as constitutive apolipoproteins of high density lipoprotein. J Biol Chem 267: 3862-3867.

52. Cases S, Smith SJ, Zheng YW, Myers HM, Lear SR, et al. (1998) Identification of a gene encoding an acyl CoA:diacylglycerol acyltransferase, a key enzyme in triacylglycerol synthesis. Proc Natl Acad Sci U S A 95: 13018-13023.

53. Cases S, Stone SJ, Zhou P, Yen E, Tow B, et al. (2001) Cloning of DGAT2, a second mammalian diacylglycerol acyltransferase, and related family members. J Biol Chem 276: 38870-38876.

54. Giller T, Buchwald P, Blum-Kaelin D, Hunziker W (1992) Two novel human pancreatic lipase related proteins, hPLRP1 and hPLRP2. Differences in colipase dependence and in lipase activity. J Biol Chem 267: 16509-16516.

55. Jenkins CM, Mancuso DJ, Yan W, Sims HF, Gibson B, et al. (2004) Identification, cloning, expression, and purification of three novel human calcium-independent phospholipase A2 family members possessing triacylglycerol lipase and acylglycerol transacylase activities. J Biol Chem 279: 48968-48975.

56. Kumashiro N, Yoshimura T, Cantley JL, Majumdar SK, Guebre-Egziabher F, et al. (2012) Role of patatin-like phospholipase domain-containing 3 on lipid-induced hepatic steatosis and insulin resistance in rats. Hepatology.

57. Mao C, Obeid LM (2008) Ceramidases: regulators of cellular responses mediated by ceramide, sphingosine, and sphingosine-1-phosphate. Biochim Biophys Acta 1781: 424-434.

58. Yoshimoto T, Yamamoto Y, Arakawa T, Suzuki H, Yamamoto S, et al. (1990) Molecular cloning and expression of human arachidonate 12-lipoxygenase. Biochem Biophys Res Commun 172: 1230-1235.

59. Heisterkamp N, Rajpert-De Meyts E, Uribe L, Forman HJ, Groffen J (1991) Identification of a human gamma-glutamyl cleaving enzyme related to, but distinct from, gamma-glutamyl transpeptidase. Proc Natl Acad Sci U S A 88: 6303-6307.

60. Sneddon AA, Wu HC, Farquharson A, Grant I, Arthur JR, et al. (2003) Regulation of selenoprotein GPx4 expression and activity in human endothelial cells by fatty acids, cytokines and antioxidants. Atherosclerosis 171: 57-65.

61. Sonoda H, Aoki J, Hiramatsu T, Ishida M, Bandoh K, et al. (2002) A novel phosphatidic acid-selective phospholipase A1 that produces lysophosphatidic acid. J Biol Chem 277: 34254-34263.

62. Han GS, Wu WI, Carman GM (2006) The Saccharomyces cerevisiae Lipin homolog is a Mg2+-dependent phosphatidate phosphatase enzyme. J Biol Chem 281: 9210-9218.

63. Shindou H, Hishikawa D, Nakanishi H, Harayama T, Ishii S, et al. (2007) A single enzyme catalyzes both platelet-activating factor production and membrane biogenesis of inflammatory cells. Cloning and characterization of acetyl-CoA:LYSO-PAF acetyltransferase. J Biol Chem 282: 6532-6539.

64. Fullerton MD, Hakimuddin F, Bakovic M (2007) Developmental and metabolic effects of disruption of the mouse CTP:phosphoethanolamine cytidylyltransferase gene (Pcyt2). Mol Cell Biol 27: 3327-3336.

65. Volinia S, Dhand R, Vanhaesebroeck B, MacDougall LK, Stein R, et al. (1995) A human phosphatidylinositol 3-kinase complex related to the yeast Vps34p-Vps15p protein sorting system. EMBO J 14: 3339-3348.

66. Zhou J, Saba JD (1998) Identification of the first mammalian sphingosine phosphate lyase gene and its functional expression in yeast. Biochem Biophys Res Commun 242: 502-507.

67. Lee WH, Lukacik P, Guo K, Ugochukwu E, Kavanagh KL, et al. (2009) Structure-activity relationships of human AKR-type oxidoreductases involved in bile acid synthesis: AKR1D1 and AKR1C4. Mol Cell Endocrinol 301: 199-204.

68. Russell DW (2003) The enzymes, regulation, and genetics of bile acid synthesis. Annu Rev Biochem 72: 137-174.

69. Taskinen JP, van Aalten DM, Knudsen J, Wierenga RK (2007) High resolution crystal structures of unliganded and liganded human liver ACBP reveal a new mode of binding for the acyl-CoA ligand. Proteins 66: 229-238.

70. Yamanashi Y, Takada T, Yoshikado T, Shoda JI, Suzuki H (2011) NPC2 Regulates Biliary Cholesterol Secretion via Stimulation of ABCG5/G8-Mediated Cholesterol Transport. Gastroenterology.

71. Radominska A, Comer KA, Zimniak P, Falany J, Iscan M, et al. (1990) Human liver steroid sulphotransferase sulphates bile acids. Biochem J 272: 597-604.

72. Yamauchi T, Kamon J, Ito Y, Tsuchida A, Yokomizo T, et al. (2003) Cloning of adiponectin receptors that mediate antidiabetic metabolic effects. Nature 423: 762-769.

73. Semple RK, Sleigh A, Murgatroyd PR, Adams CA, Bluck L, et al. (2009) Postreceptor insulin resistance contributes to human dyslipidemia and hepatic steatosis. J Clin Invest 119: 315-322.

74. Moreau K, Dizin E, Ray H, Luquain C, Lefai E, et al. (2006) BRCA1 affects lipid synthesis through its interaction with acetyl-CoA carboxylase. J Biol Chem 281: 3172-3181.

75. Inoue Y, Inoue J, Lambert G, Yim SH, Gonzalez FJ (2004) Disruption of hepatic C/EBPalpha results in impaired glucose tolerance and age-dependent hepatosteatosis. J Biol Chem 279: 44740-44748.

76. Wang Y, Zhao L, Smas C, Sul HS (2010) Pref-1 interacts with fibronectin to inhibit adipocyte differentiation. Mol Cell Biol 30: 3480-3492.

77. Affholter JA, Hsieh CL, Francke U, Roth RA (1990) Insulin-degrading enzyme: stable expression of the human complementary DNA, characterization of its protein product, and chromosomal mapping of the human and mouse genes. Mol Endocrinol 4: 1125-1135.

78. Souza SC, Chau MD, Yang Q, Gauthier MS, Clairmont KB, et al. (2011) Atrial natriuretic peptide regulates lipid mobilization and oxygen consumption in human adipocytes by activating AMPK. Biochem Biophys Res Commun 410: 398-403.

79. Goldstein JL, DeBose-Boyd RA, Brown MS (2006) Protein sensors for membrane sterols. Cell 124: 35-46.

80. Neumeier M, Weigert J, Schaffler A, Weiss T, Kirchner S, et al. (2005) Regulation of adiponectin receptor 1 in human hepatocytes by agonists of nuclear receptors. Biochem Biophys Res Commun 334: 924-929.

81. Horton JD, Shah NA, Warrington JA, Anderson NN, Park SW, et al. (2003) Combined analysis of oligonucleotide microarray data from transgenic and knockout mice identifies direct SREBP target genes. Proc Natl Acad Sci U S A 100: 12027-12032.

82. Porsch-Ozcurumez M, Langmann T, Heimerl S, Borsukova H, Kaminski WE, et al. (2001) The zinc finger protein 202 (ZNF202) is a transcriptional repressor of ATP binding cassette transporter A1 (ABCA1) and ABCG1 gene expression and a modulator of cellular lipid efflux. J Biol Chem 276: 12427-12433.

83. Akiyama S, Endo H, Inohara N, Ohta S, Kagawa Y (1994) Gene structure and cell type-specific expression of the human ATP synthase alpha subunit. Biochim Biophys Acta 1219: 129-140.

84. Ohta S, Kagawa Y (1986) Human F1-ATPase: molecular cloning of cDNA for the beta subunit. J Biochem 99: 135-141.

85. Janssen U, Fink T, Lichter P, Stoffel W (1994) Human mitochondrial 3,2-trans-enoyl-CoA isomerase (DCI): gene structure and localization to chromosome 16p13.3. Genomics 23: 223-228.

86. Ziouzenkova O, Orasanu G, Sharlach M, Akiyama TE, Berger JP, et al. (2007) Retinaldehyde represses adipogenesis and diet-induced obesity. Nat Med 13: 695-702.

87. Greenfield NJ, Pietruszko R (1977) Two aldehyde dehydrogenases from human liver. Isolation via affinity chromatography and characterization of the isozymes. Biochim Biophys Acta 483: 35-45.

88. Hsu LC, Chang WC, Hiraoka L, Hsieh CL (1994) Molecular cloning, genomic organization, and chromosomal localization of an additional human aldehyde dehydrogenase gene, ALDH6. Genomics 24: 333-341.

89. Huang DY, Ichikawa Y (1994) Two different enzymes are primarily responsible for retinoic acid synthesis in rabbit liver cytosol. Biochem Biophys Res Commun 205: 1278-1283.

90. Haeseleer F, Huang J, Lebioda L, Saari JC, Palczewski K (1998) Molecular characterization of a novel short-chain dehydrogenase/reductase that reduces all-trans-retinal. J Biol Chem 273: 21790-21799.

91. Deisenroth C, Itahana Y, Tollini L, Jin A, Zhang Y (2011) p53-Inducible DHRS3 is an endoplasmic reticulum protein associated with lipid droplet accumulation. J Biol Chem 286: 28343-28356.

92. Seehafer JG, Slupsky JR, Tang SC, Shaw AR (1988) The functional cell surface glycoprotein CD9 is distinguished by being the major fatty acid acylated and a major iodinated cell-surface component of the human platelet. Biochim Biophys Acta 952: 92-100.

93. Lancellotti S, De Cristofaro R (2009) Congenital prothrombin deficiency. Semin Thromb Hemost 35: 367-381.

94. Santagata S, Boggon TJ, Baird CL, Gomez CA, Zhao J, et al. (2001) G-protein signaling through tubby proteins. Science 292: 2041-2050.

95. Keppler OT, Hinderlich S, Langner J, Schwartz-Albiez R, Reutter W, et al. (1999) UDP-GlcNAc 2-epimerase: a regulator of cell surface sialylation. Science 284: 1372-1376.

96. Takahashi N, Takahashi Y, Putnam FW (1985) Complete amino acid sequence of human hemopexin, the heme-binding protein of serum. Proc Natl Acad Sci U S A 82: 73-77.

97. Miller YI, Smith A, Morgan WT, Shaklai N (1996) Role of hemopexin in protection of low-density lipoprotein against hemoglobin-induced oxidation. Biochemistry 35: 13112-13117.

98. Wu L, Einstein M, Geissler WM, Chan HK, Elliston KO, et al. (1993) Expression cloning and characterization of human 17 beta-hydroxysteroid dehydrogenase type 2, a microsomal enzyme possessing 20 alpha-hydroxysteroid dehydrogenase activity. J Biol Chem 268: 12964-12969.

99. Jiao H, Arner P, Hoffstedt J, Brodin D, Dubern B, et al. (2011) Genome wide association study identifies KCNMA1 contributing to human obesity. BMC Med Genomics 4: 51.

100. Merkulov S, Zhang WM, Komar AA, Schmaier AH, Barnes E, et al. (2008) Deletion of murine kininogen gene 1 (mKng1) causes loss of plasma kininogen and delays thrombosis. Blood 111: 1274-1281.

101. Petit MM, Mols R, Schoenmakers EF, Mandahl N, Van de Ven WJ (1996) LPP, the preferred fusion partner gene of HMGIC in lipomas, is a novel member of the LIM protein gene family. Genomics 36: 118-129.

102. Klomp LW, de Koning TJ, Malingre HE, van Beurden EA, Brink M, et al. (2000) Molecular characterization of 3-phosphoglycerate dehydrogenase deficiency--a neurometabolic disorder associated with reduced L-serine biosynthesis. Am J Hum Genet 67: 1389-1399.

103. Doherty MJ, Young PR, Cohen PT (1996) Amino acid sequence of a novel protein phosphatase 1 binding protein (R5) which is related to the liver- and muscle-specific glycogen binding subunits of protein phosphatase 1. FEBS Lett 399: 339-343.

104. Camp LA, Verkruyse LA, Afendis SJ, Slaughter CA, Hofmann SL (1994) Molecular cloning and expression of palmitoyl-protein thioesterase. J Biol Chem 269: 23212-23219.

105. Beck BD, Park SJ, Lee YJ, Roman Y, Hromas RA, et al. (2008) Human Pso4 is a metnase (SETMAR)-binding partner that regulates metnase function in DNA repair. J Biol Chem 283: 9023-9030.

106. Cho SY, Shin ES, Park PJ, Shin DW, Chang HK, et al. (2007) Identification of mouse Prp19p as a lipid droplet-associated protein and its possible involvement in the biogenesis of lipid droplets. J Biol Chem 282: 2456-2465.

107. Canessa CM, Schild L, Buell G, Thorens B, Gautschi I, et al. (1994) Amiloride-sensitive epithelial Na+ channel is made of three homologous subunits. Nature 367: 463-467.

108. Guo X, Cheng S, Taylor KD, Cui J, Hughes R, et al. (2005) Hypertension genes are genetic markers for insulin sensitivity and resistance. Hypertension 45: 799-803.

109. Lane DA, Olds RR, Thein SL (1992) Antithrombin and its deficiency states. Blood Coagul Fibrinolysis 3: 315-341.

110. Loskutoff DJ, Sawdey M, Mimuro J (1989) Type 1 plasminogen activator inhibitor. Prog Hemost Thromb 9: 87-115.

111. Nykjaer A, Petersen CM, Moller B, Jensen PH, Moestrup SK, et al. (1992) Purified alpha 2-macroglobulin receptor/LDL receptor-related protein binds urokinase.plasminogen activator inhibitor type-1 complex. Evidence that the alpha 2-macroglobulin receptor mediates cellular degradation of urokinase receptor-bound complexes. J Biol Chem 267: 14543-14546.

112. Asahina M, Haruyama W, Ichida Y, Sakamoto M, Sato M, et al. (2009) Identification of SMEK2 as a candidate gene for regulation of responsiveness to dietary cholesterol in rats. J Lipid Res 50: 41-46.

113. Ahn AH, Yoshida M, Anderson MS, Feener CA, Selig S, et al. (1994) Cloning of human basic A1, a distinct 59-kDa dystrophin-associated protein encoded on chromosome 8q23-24. Proc Natl Acad Sci U S A 91: 4446-4450.

114. Adachi J, Kumar C, Zhang Y, Mann M (2007) In-depth analysis of the adipocyte proteome by mass spectrometry and bioinformatics. Mol Cell Proteomics 6: 1257-1273.

115. Johnson JD, Muhonen WW, Lambeth DO (1998) Characterization of the ATP- and GTP-specific succinyl-CoA synthetases in pigeon. The enzymes incorporate the same alpha-subunit. J Biol Chem 273: 27573-27579.

116. Sousa MM, Berglund L, Saraiva MJ (2000) Transthyretin in high density lipoproteins: association with apolipoprotein A-I. J Lipid Res 41: 58-65.

117. Buxbaum JN, Reixach N (2009) Transthyretin: the servant of many masters. Cell Mol Life Sci 66: 3095-3101.

118. Weissglas-Volkov D, Pajukanta P (2010) Genetic causes of high and low serum HDL-cholesterol. J Lipid Res 51: 2032-2057.

119. Sato S, Fujita N, Tsuruo T (2002) Regulation of kinase activity of 3-phosphoinositide-dependent protein kinase-1 by binding to 14-3-3. J Biol Chem 277: 39360-39367.
